# Silica-Coated Micrometer-Sized Latex Particles

**DOI:** 10.1021/acs.langmuir.3c00227

**Published:** 2023-03-31

**Authors:** O. Norvilaite, C. Lindsay, P. Taylor, S. P. Armes

**Affiliations:** † Dainton Building, Department of Chemistry, University of Sheffield, Brook Hill, Sheffield, South Yorkshire S3 7HF, UK; ‡ Syngenta, Jealott’s Hill International Research Centre, Bracknell, Berkshire RG42 6EY, UK

## Abstract

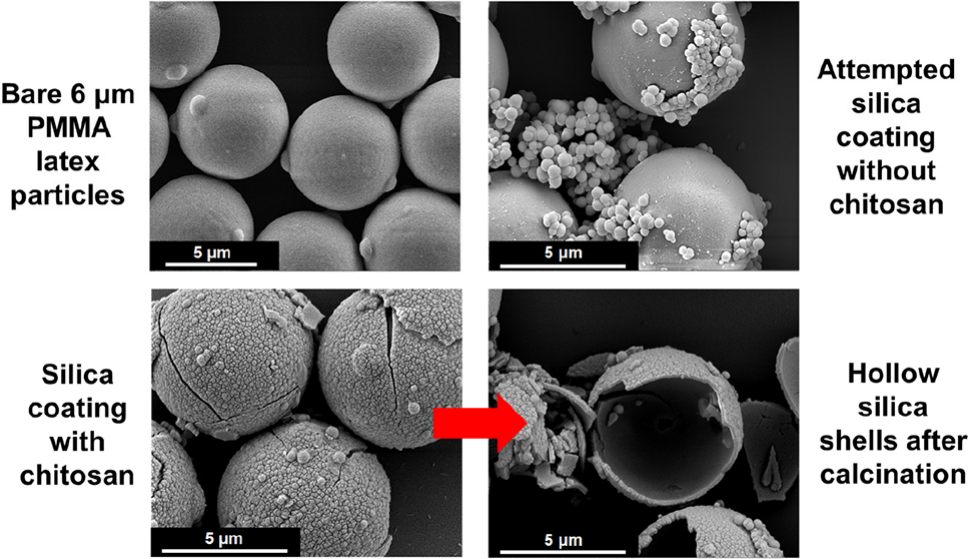

A series of silica-coated micrometer-sized poly(methyl methacrylate) latex particles are prepared using a Stöber silica deposition protocol that employs tetraethyl orthosilicate (TEOS) as a soluble silica precursor. Given the relatively low specific surface area of the latex particles, silica deposition is best conducted at relatively high solids to ensure a sufficiently high surface area. Such conditions aid process intensification. Importantly, physical adsorption of chitosan onto the latex particles prior to silica deposition minimizes secondary nucleation and promotes the formation of silica shells: in the absence of chitosan, well-defined silica overlayers cannot be obtained. Thermogravimetry studies indicate that silica formation is complete within a few hours at 20 °C regardless of the presence or absence of chitosan. Kinetic data obtained using this technique suggest that the adsorbed chitosan chains promote surface deposition of silica onto the latex particles but do not catalyze its formation. Systematic variation of the TEOS/latex mass ratio enables the mean silica shell thickness to be tuned from 45 to 144 nm. Scanning electron microscopy (SEM) studies of silica-coated latex particles after calcination at 400 °C confirm the presence of hollow silica particles, which indicates the formation of relatively smooth (albeit brittle) silica shells under optimized conditions. Aqueous electrophoresis and X-ray photoelectron spectroscopy studies are also consistent with latex particles coated in a uniform silica overlayer. The silica deposition formulation reported herein is expected to be a useful generic strategy for the efficient coating of micrometer-sized particles at relatively high solids.

## Introduction

There are many examples of particles with a well-defined core-shell morphology in the literature. In many cases, both the core and the shell are polymeric. For example, it is well-known that judicious selection of copolymer cores and shells with differing glass transition temperatures lead to useful paints and coatings.^1−5^ Other examples include conducting polymer-coated latex particles,^6−10^ which have been employed as synthetic mimics for understanding the behavior of organic-rich micro-meteorites.^11,12^ Precious metal deposition onto conducting polymer-coated polystyrene latex particles produces heterogeneous catalysts, which have been evaluated for Suzuki coupling reactions**.**^13,14^

There are also many examples in which one component (i.e., core or shell) is polymeric and the other component (shell or core) comprises an inorganic material. For example, metal shells have been deposited onto poly(methyl methacrylate) latexes,^15^ while titania-coated polystyrene latexes have been evaluated as a lightweight filler material or for controlled drug release applications.^16^ Similarly, polymer-silica core-shell particles^17−20^ enable the design of tough, dirt-shedding nanocomposite films^21−23^ and are an important intermediate in the design of state-of-the-art anti-reflective coatings for solar panels.^24,25^ Such systems can also serve as useful model systems for understanding visible Mie scattering, which can lead to structural color.^26,27^ In this context, various strategies have been examined for the deposition of silica onto latex particles.^28,29^ In some cases, silica nanoparticles have simply been physically adsorbed onto sterically stabilized latexes to produce a particulate shell.^30^ More typically, a soluble silica precursor is utilized to form a smooth uniform overlayer via in situ hydrolysis-condensation reactions. Suitable silica precursors include tetraethyl orthosilicate (TEOS),^31−51^ tetramethyl orthosilicate (TMOS),^31,52−56^ and sodium silicate.^20,33,34,57−60^

Sodium silicate is a low-cost silica precursor that is often used for wholly aqueous formulations.^20,59,61−63^ For such syntheses, Si(OH)_4_ can be generated using either a cation exchange resin or by lowering the solution pH to pH 2. Alternatively, base-catalyzed condensation may be employed to produce a colloidal silica sol at pH 7–10.^20,57−60^ Although sodium silicate is a greener and more cost-effective soluble silica precursor,^28,34^ the growth of a silica shell is strongly pH-dependent and sometimes difficult to control. One study compared silica deposition onto polystyrene latex particles using either sodium silicate or TEOS as precursors.^34^ Sodium silicate formulations led to patchy, non-uniform overlayers and tended to produce aggregates. On the other hand, TEOS led to relatively uniform silica coatings with minimal particle aggregation.

As discussed above, silica deposition protocols can be either acid- or base-catalyzed. Acid-catalyzed reactions produce weakly crosslinked gel networks.^62,64^ In contrast, colloidal Stöber silica sols can be obtained via base catalysis, which typically involves using ammonia in an ethanol-rich aqueous solution.^66,62^ In principle, the relative rates of hydrolysis and condensation depend on the ammonia concentration, and the silica overlayer thickness can be adjusted by systematically varying the TEOS concentration.^66^ This reagent is water-immiscible, so ethanol is used as a co-solvent to ensure its solubilization. Alternatively, ethanol-free formulations utilizing a suitable surfactant to solubilize the TEOS have been reported.^41,48,51^

TMOS is water-miscible and hence significantly more reactive than TEOS with respect to the initial hydrolysis step, which leads to the rapid generation of water-soluble Si(OH)_4_ species. Thus TMOS is sometimes employed without using any ethanol co-solvent.^66,67^ Some literature formulations use a binary mixture of TMOS and TEOS, or a combination of 3-(glycidyloxypropyl)trimethoxysilane (GPTMS) with TEOS, or trimethylethoxysilane (TMES) with TMOS.^31,54,68^ Unfortunately, TMOS hydrolysis produces methanol, which is much more toxic than the ethanol by-product generated via TEOS hydrolysis. Moreover, the latter reagent is more cost-effective than TMOS, so it is usually preferred for silica deposition formulations despite its lower reactivity. Taking into account the extensive (and sometimes conflicting) literature outlined above, we decided to employ TEOS as a soluble silica precursor for the development of a facile silica deposition protocol.

Most silica-coating protocols reported in the literature are restricted to colloidal particles (<1 μm diameter), and the target silica overlayer thickness is usually no more than 100 nm. However, hydrolysis of TEOS using aqueous HCl followed by ammonia-catalyzed condensation has been used to coat polyurea microcapsules of 57–328 μm diameter to produce a polyurea/silica hybrid overlayer of 1–8 μm thickness.^69^ Similarly, carbon nanofibers (mean fiber diameter = 20–150 nm and length = 10–30 μm) have been coated with silica via acid hydrolysis–condensation of TEOS.^70^ The same team found that the base-catalyzed polycondensation of commercial pre-hydrolyzed ethyl silicate produced a higher surface coverage within shorter reaction times.^70^ Nevertheless, the vast majority of such studies utilize relatively small latex particles (60 to 1000 nm diameter), and silica deposition normally involves rather dilute solution conditions (typically less than 1% w/w solids).^36,39−49,52,58,60,71^ In particular, there are very few studies focused on coating relatively large latex particles^37,38,59^ with thin silica shells in semi-concentrated solution.^42,51,71^

**Scheme 1:**
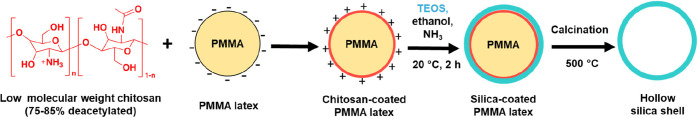
Schematic Representation of the Deposition of a Relatively Thin Silica Overlayer onto Micrometer-Sized PMMA Latex Particles Prior adsorption of chitosan onto the latex particles is important because it minimizes secondary nucleation (see main text for further details).

Herein, we report the preparation and characterization of silica-coated poly(methyl methacrylate) (PMMA) latex particles. These latex particles are commercially available, highly cross-linked, lie in the micrometer size range and have reasonably narrow size distributions. Given their relatively low specific surface area (<1 m^2^ g^–1^), silica deposition is best conducted at high solids (e.g., 8.2–23.5% w/w) rather than in dilute solution because this provides sufficient surface area to minimize secondary nucleation. To further reduce this well-documented problem, a naturally occurring cationic biopolymer (chitosan) is physically adsorbed onto the surface of the latex particles prior to silica deposition (see Scheme 1). The primary amine groups on the chitosan chains promote surface deposition of the silica, which is generated using TEOS as a soluble silica precursor.^65^ The resulting silica-coated latex particles are characterized using scanning electron microscopy, optical microscopy, thermogravimetry, laser diffraction, aqueous electrophoresis, and X-ray photoelectron spectroscopy (XPS). This chitosan-coated latex system is expected to be a useful model for understanding and optimizing silica deposition onto other micrometer-sized particles using a potentially scalable process-intensive formulation.

## Experimental Section

### Materials

Micrometer-sized crosslinked poly(methyl methacrylate) latexes with nominal diameters of 6, 10, or 15 μm and linear polystyrene latex with a nominal diameter of 20 μm were purchased from Microbeads (Skedsmokorset, Norway). Low molecular weight chitosan and tetraethyl orthosilicate (TEOS; 98% purity) were purchased from Sigma-Aldrich (UK). Concentrated ammonium hydroxide solution (28%) was purchased from Alfa Aesar (UK). Absolute ethanol (≥99.8%, HPLC grade) and methanol (≥99.8%, HPLC grade) were purchased from Fisher (UK). Glacial acetic acid and tetrahydrofuran (THF) (HPLC grade) were purchased from VWR (UK). Deionized water was used for all experiments.

#### Adsorption of Chitosan onto PMMA Latex Particles

Chitosan (2.000 g) was dissolved in 0.1 M acetic acid (100 mL) to produce a 20 g dm^–3^ aqueous stock solution, which was magnetically stirred overnight to ensure complete dissolution. For route A, 6 μm PMMA latex particles (5.000 g) were dispersed in deionized water (45.000 g) with the aid of magnetic stirring for 5 min. Then one droplet of chitosan stock solution (104 μL, 2.075 mg chitosan) was added to this 10% w/w latex suspension. For route B, a 104 μL droplet of the same 20 g dm^–3^ chitosan stock solution (2.075 mg chitosan) was diluted with deionized water (45.730 g), followed by stepwise addition of dry 6 μm PMMA latex particles (5.000 g; approximately 1 g per portion), with vigorous mixing between each addition to afford a ∼10% w/w latex suspension at pH 3.5–4.0. After mixing this suspension overnight using a roller mixer at 20 °C, the chitosan-coated PMMA latex particles were isolated by freeze-drying overnight. For the 10 and 15 μm PMMA latex particles, the same protocol was used, but the mass of chitosan was adjusted to either 1.250 or 0.825 mg, respectively, to account for the lower specific surface area of each latex.

#### Variation of the Chitosan/PMMA Mass Ratio

PMMA latex (6 μm, 50.0 mg) was dispersed in deionized water (4.792–4.958 mL). Then, 0.042–0.208 mL of a 0.20 g dm^–3^ chitosan stock solution in 0.1 M acetic acid was added to afford a 1.0% w/w suspension at pH 3.5–4.0, with a chitosan/PMMA mass ratio ranging from 0.17 × 10^–3^ to 0.83 × 10^–3^. Each suspension was placed on a roller mixer overnight at 20 °C to ensure maximum chitosan adsorption.

#### Silica Deposition onto Chitosan-Coated PMMA Latex Particles

Chitosan-coated 6 μm PMMA latex (200 mg; 0.166 m^2^) was dispersed in 28% ammonium hydroxide (0.409 mL) with the aid of magnetic stirring for 5 min. Then, a 1.858 mL aliquot of a 10% v/v ethanolic solution of TEOS (0.173 g, 0.830 mmol TEOS; target silica thickness = 148 nm; see entry 8 in Table 1) was added to the PMMA latex suspension and stirred for 2 h at 20 °C. The resulting silica-coated PMMA latex particles were sedimented via centrifugation (5000 rpm for 10 min; Beckman Coulter Avanti J-25 centrifuge), followed by redispersion first in ethanol (three times) and then in methanol (once). After decanting the final supernatant, the silica-coated PMMA latex particles were allowed to dry at 20 °C overnight. The mass of 6 μm PMMA latex was held constant at 200 mg while the target silica mass loading was systematically lowered, which reduces the target silica shell thickness from 148 to 46 nm (see eq 1). The required mass of TEOS (e.g., 50 mg) was calculated for the desired silica mass loading (e.g., 20%). The volume of 28% aqueous ammonia solution was adjusted to maintain a constant TEOS/ammonia molar ratio of 0.137 in each case. Silica deposition experiments involving 10 or 15 μm PMMA latex particles were conducted at the same total surface area as that employed for the 6 μm PMMA latex (see above). The target silica mass loadings for the 10 and 15 μm PMMA latexes were 13.1 and 9.1%, which correspond to target silica overlayer thicknesses of 152 and 153 nm, respectively.

**Table 1:** Summary of the Effect of Varying the Target Silica Loading and Target Silica Shell Thickness when Preparing Silica-Coated Micrometer-Sized PMMA Latex Particles in the Presence or Absence of Chitosan Using TEOS as Soluble Silica Precursor and a Stöber-Type Formulation at 20 °C^b^

				silica loading (%)		shell thickness (nm)	
latex diameter (μm)	latex concentration (w/v%)	10% v/v TEOS in ethanol (ml)	28% ammonia (μL)	target	final	target	final
6	23.5	0.559	123	7.0	6.8	46	45
	17.1	0.826	182	10.0	9.7	68	66
	14.3	1.014	223	12.0	11.8	83	81
	12.1	1.210	266	14.0	13.9	98	98
	10.6	1.416	312	15.9	15.7	114	112
	9.3	1.632	359	18.0	17.7	131	129
	8.2^a^	1.858	409	20.0	20.0	no shell	no shell
	8.2	1.858	409	19.9	19.5	148	144
10	13.6	1.858	409	13.1	13.0	152	151
15	18.6	1.858	409	9.1	9.1	153	153

a Control experiment using bare 6 μm PMMA latex (absence of any chitosan).

b All syntheses were conducted at a constant latex surface area.

Silica deposition was also performed using 20 μm linear polystyrene latex particles. The non-crosslinked nature of such particles enabled hollow silica shells to be obtained via polystyrene dissolution in hot THF (see Supporting Information for further details). Removal of the latex cores without recourse to calcination avoids further densification via further silanol reactions at elevated temperature. Hence measurement of the density of the remaining silica shells via helium pycnometry should provide a more reliable density for the silica overlayer, which is deposited onto the latex particles at 20 °C. Accordingly, the densities of chitosan-coated polystyrene latex, silica-coated polystyrene latex and hollow silica shells are summarized in Table S1, and the corresponding FT-IR spectra are shown in Figure S1. SEM images recorded for the silica-coated polystyrene latex and the remaining hollow silica shells following dissolution of the polystyrene latex cores are shown in Figure S2.

#### Control Experiment Using Bare 6 μm PMMA Latex

Silica deposition onto bare 6 μm PMMA latex particles was also attempted in the absence of any chitosan using the above protocol. In this case, the target silica shell thickness was 148 nm.

#### Optical Microscopy

PMMA latex particles were imaged before and after silica deposition (and also after calcination at 500 °C) using a Cole Parmer microscope equipped with a MoticamBTW camera and an LCD tablet. ImageJ 1.53k software was employed for particle size analysis.

#### Scanning Electron Microscopy

SEM images were recorded using an FEI Inspect F field emission scanning electron microscope at an acceleration voltage of 5 kV. All samples were prepared by drying dilute aqueous suspensions onto silicon wafer chips. Sample-loaded silicon wafers were mounted onto aluminum stubs using adhesive carbon tabs. Silver paint was applied to two edges of the mounted silicon wafers followed by sputter coating to produce a 5 nm gold overlayer, which minimizes sample-charging. ImageJ 1.53k software was employed for silica shell thickness measurements.

#### Laser Diffraction

PMMA latexes were analyzed before and after chitosan adsorption and silica deposition to determine their mean particle size using a Malvern Mastersizer 3000 instrument equipped with a Hydro EV wet dispersion unit at 2000 rpm, a red HeNe laser (λ = 633 nm), and an LED blue light source (λ = 470 nm). The median particle diameter, D50, and volume-average diameter, D[4,3], were averaged over five measurements. The instrument was rinsed three times with deionized water between measurements to prevent cross-contamination.

#### Aqueous Electrophoresis

A Malvern Zetasizer Nano ZS instrument was used to analyze 6 μm PMMA latex particles before and after chitosan adsorption and silica deposition. The Smoluchowski approximation was applied to calculate zeta potentials using the Henry equation. PMMA latex suspensions were diluted to 0.05% w/w using 1 mM KCl as background electrolyte. The pH of each suspension was monitored using a pH probe and adjusted as required using either 0.5 M HCl or 0.5 M NaOH. Each measurement was performed in triplicate to obtain a mean value.

#### Thermogravimetry

Silica-coated PMMA latex particles were heated up to 500 °C at a heating rate of 10 °C min^–1^ under air using a Q500 thermogravimetric analyzer (TA Instruments). This leads to complete pyrolysis of the PMMA, which enables determination of the original silica mass loading on the latex particles.

#### Helium Pycnometry

Solid-state densities were determined using a calibrated Micromeritics AccuPyc II 1345 helium pycnometer operating at 20 °C.

#### X-ray Photoelectron Spectroscopy

Bare, chitosan-coated, and silica-coated PMMA latex particles were analyzed using a Kratos Axis Supra X-ray photoelectron spectrometer. Chitosan and silica (formed in the presence of chitosan but the absence of any latex) were also analyzed as reference materials. Step sizes of 1.0 and 0.1 eV were used to record survey spectra and high resolution spectra, respectively. In each case, spectra were recorded from at least two separate areas and analyzed using Casa XPS software (UK). All binding energies were calibrated with respect to the saturated hydrocarbon C1s signal at 285.0 eV.

#### FT-IR Spectroscopy

FT-IR spectra were recorded for chitosan-coated polystyrene latex, silica-coated polystyrene latex, and the hollow silica shells that remain following dissolution of the linear polystyrene chains using THF. These spectra were recorded using a PerkinElmer Spectrum 100 FT-IR spectrophotometer equipped with a Universal Attenuated Total Reflectance (UATR) module. The spectra were obtained between 4000 and 400 cm^–1^ at a spectral resolution of 4 cm^–1^; 12 scans were averaged per spectrum.

## Results and Discussion

**Figure 1:**
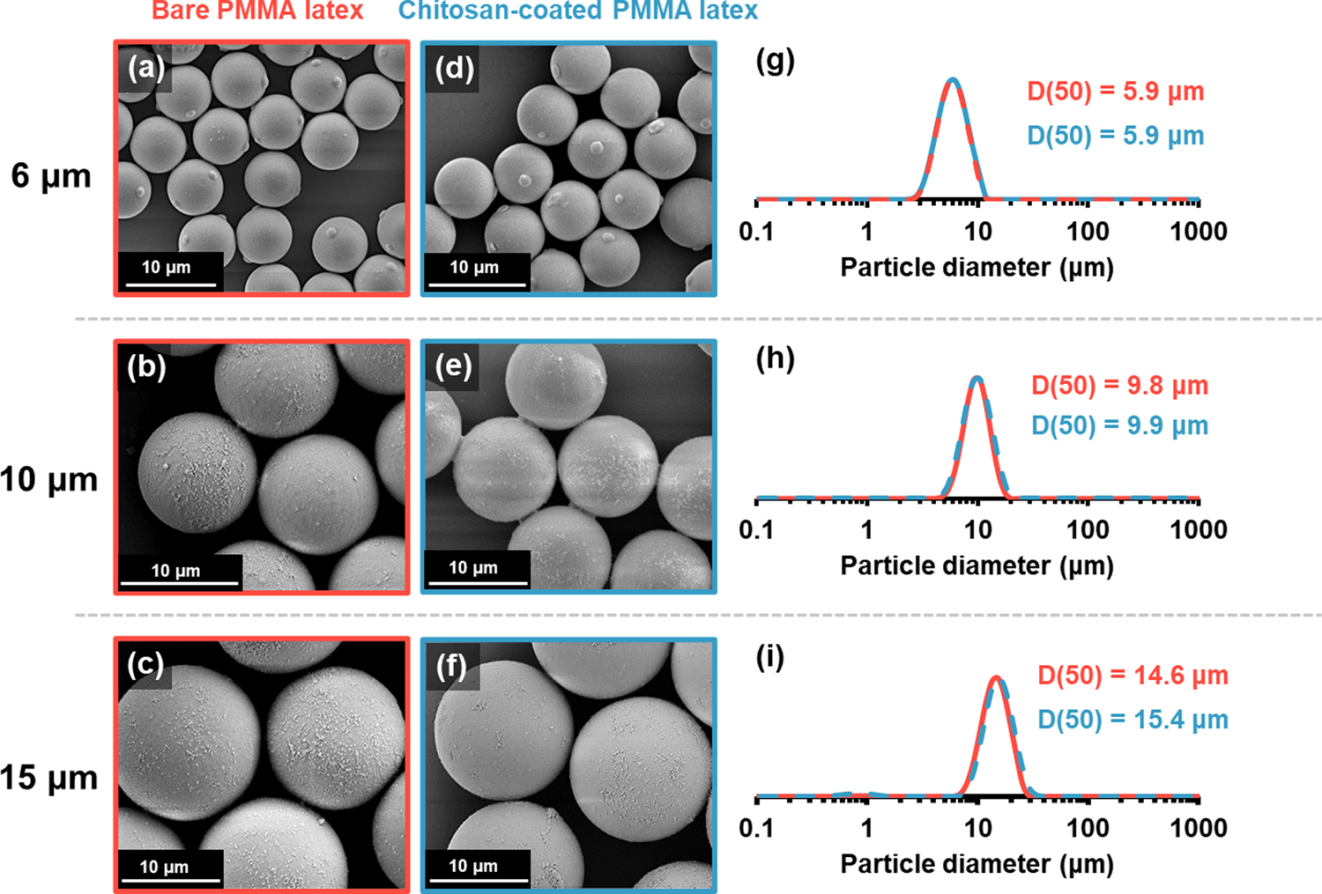
SEM images and laser diffraction particle size distributions obtained for PMMA latex particles before (red) and after (blue) adsorption of chitosan. In this set of experiments, the latex particles were gradually added to an acidic aqueous solution of chitosan to minimize bridging flocculation.

The three latexes used in this study were commercially sourced micrometer-sized crosslinked PMMA particles with nominal diameters of 6, 10, or 15 μm. Scanning electron microscopy (SEM) studies confirmed their spherical morphology and indicated a relatively smooth, featureless surface in each case, although a few spherical nodules were visible at the surface of the 6 μm latex particles (see Figure 1). Particle size distributions were assessed using laser diffraction, which indicated volume-average (D[4,3]) diameters of 6.1, 10.1, and 14.9 μm, respectively. The corresponding median (D(50)) diameters were 5.9, 9.8, and 14.6 μm, while the spans were 0.75, 0.66, and 0.72, respectively.

**Figure 2:**
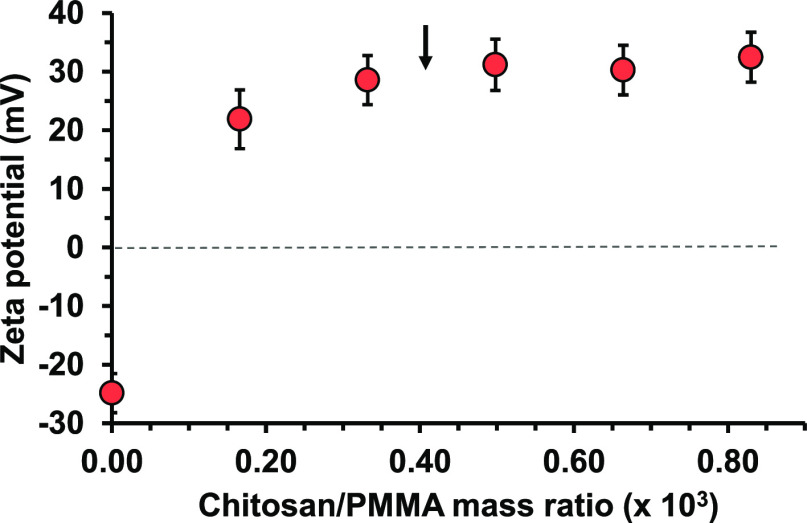
Change in zeta potential (determined at pH 4.8) when varying the chitosan/PMMA mass ratio for the adsorption of chitosan onto 6 μm PMMA latex particles. The vertical black arrow indicates the chitosan/PMMA mass ratio employed for the subsequent silica deposition experiments summarized in Table 1.

According to the literature, silica deposition onto colloidal particles can be enhanced by appropriate surface functionalization.^17,58,60,72,73^ More specifically, it is known that cationic character promotes the formation of silica-coated particles.^42,43,58,74,75^ The primary objective for the present study was to deposit a silica overlayer of tunable thickness onto micrometer-sized model particles at relatively high solids while minimizing secondary nucleation. To achieve this aim, we identified chitosan as a suitable biorenewable additive that should promote surface deposition.^76,77^ In our initial experiments, this primary amine-functionalized biopolymer was physically adsorbed onto the 6 μm PMMA latex, which was selected to facilitate aqueous electrophoresis studies (the two larger latexes are prone to sedimentation during such measurements). The zeta potential of −24.9 mV recorded for the bare 6 μm PMMA latex at pH 4.8 confirmed its anionic surface character (see Figure 2). Chitosan is a highly cationic polyelectrolyte at this pH, so its electrostatic adsorption should lead to a relatively low adsorbed amount.^78^

As the chitosan/PMMA mass ratio was systematically increased, the latex zeta potential became initially less negative and then positive. According to a prior study by Williams and co-workers, the plateau zeta potential of approximately +30 mV observed for this curve (see black arrow) should correspond to maximum surface coverage of the latex particles by the chitosan.^79^ This occurred at an approximate chitosan/PMMA mass ratio of 0.0004, which suggests an adsorbed amount of chitosan of around 0.50 mg m^–2^.

A relatively low molecular weight chitosan was selected for the present study. In principle, this should prevent any possibility of bridging flocculation during its electrostatic adsorption onto micrometer-sized latex particles. In practice, addition of chitosan to the latex particles led to a discernible reduction in the degree of dispersion even for the largest latex particles, as judged by the slightly skewed particle size distributions determined by laser diffraction studies (see Figure S3). Fortunately, this problem was eliminated simply by changing the order of addition. Thus, slowly adding the latex particles to an acidic aqueous solution of chitosan led to essentially the same particle size distribution being observed as that obtained in the absence of any chitosan (see laser diffraction data in Figure 1).

**Figure 3:**
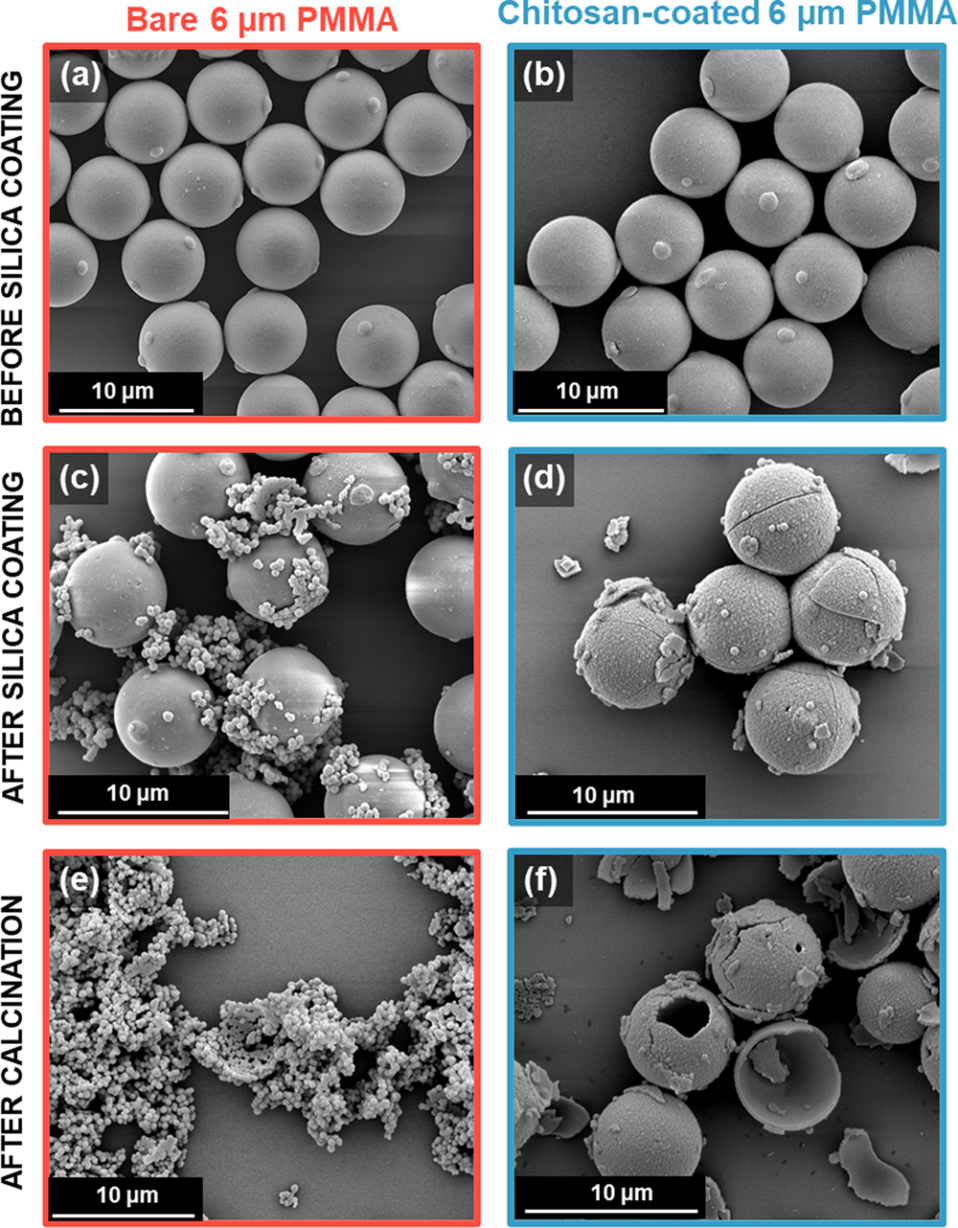
SEM images recorded for (a) the bare 6 μm PMMA latex, (b) chitosan-coated 6 μm PMMA latex, (c) attempted silica coating of PMMA latex in the absence of chitosan; (d) silica-coated PMMA latex prepared in the presence of chitosan; (e) attempted silica coating of PMMA latex in the absence of chitosan after calcination (no evidence for formation of hollow silica shells); (f) silica-coated PMMA latex prepared in the presence of chitosan after calcination (note the formation of silica hollow shells).

A series of silica coating experiments were conducted using a Stöber-type formulation in which the TEOS/latex mass ratio was systematically varied; the results are summarized in Table 1. Inspecting the SEM images shown in Figure 3, controlled silica deposition onto the bare PMMA latex clearly does not occur in the absence of chitosan. Instead, substantial secondary nucleation of silica particles is observed. In striking contrast, prior adsorption of chitosan promotes relatively uniform silica deposition at the surface of the PMMA latex particles. Optical microscopy studies support these SEM observations (see Figure S4).

**Figure 4:**
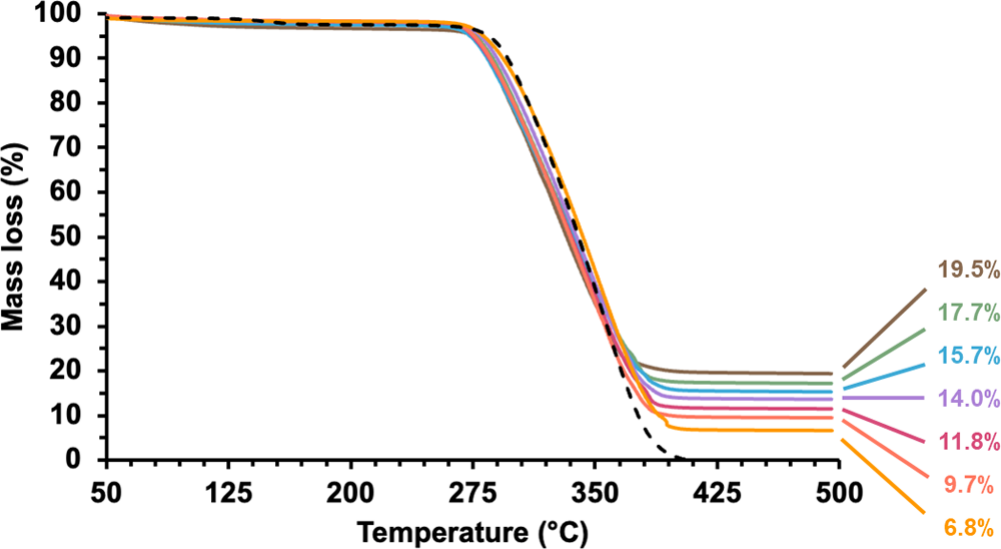
Representative thermogravimetry curves recorded for chitosan-coated 6 μm PMMA latex (black dashed line) and six examples of silica-coated 6 μm PMMA latex particles when targeting silica shell thicknesses of 46 nm (yellow curve), 68 nm (orange curve), 83 nm (red curve), 98 nm (purple curve), 114 nm (blue curve), 131 nm (green curve), and 148 nm (brown curve); see Table 1.

Thermogravimetry was used to assess the chemical composition of silica-coated latex particles. The thermal degradation of PMMA has been extensively studied: it is well-known that such methacrylic chains unzip cleanly to afford MMA monomer with few side reactions and no char formation.^80^ Indeed, for the crosslinked PMMA latex particles used herein, complete pyrolysis was achieved on heating up to 400 °C in air at a heating rate of 10 °C min^–1^ (see Figure 4). Similarly, it is well-established that silica is a refractory material that does not suffer any significant loss in mass (except for surface dehydration) under such conditions. Hence the original silica mass content of dried silica-coated latex particles can be readily determined by thermogravimetry. These TGA residues were corrected by using the % mass loss observed at 130 °C to calculate the extent of surface dehydration of the silica overlayer.

Inspecting Table 1, the actual silica mass content correlates well with the theoretical silica mass content, which is calculated from the initial TEOS mass by assuming that each gram of TEOS produces 0.288 g SiO_2_. If it is assumed that all such silica is deposited onto the latex particles (i.e., that secondary nucleation is negligible), then the mean silica shell thickness (*x*) can be calculated using eq 1, which was derived for core-shell spherical particles by Lascelles and Armes.^6^
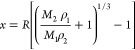
1where *R* is the mean core radius, *M*_1_ and *M*_2_ are the mass fractions of the core and shell components, and ρ_1_ and ρ_2_ are the solid-state densities of the core and shell components. Such overlayer thickness calculations require the density of the silica overlayer. According to the literature, the solid-state density of silica produced when using TEOS or TMOS typically ranges from 1.60 to 2.00 g cm^–3^ owing to the presence of unreacted alkoxy groups within the network.^81−84^ In contrast, silica generated from sodium silicate has a somewhat higher density of 2.00–2.20 g cm^–3^.^57,85^ To obtain a reliable density for the silica overlayer, a silica deposition control experiment was performed using a chitosan-coated 20 μm polystyrene latex (see Figure S2, the Experimental Section, and the Supporting Information for further details). Unlike the highly crosslinked PMMA latexes, this latex comprises linear chains that can be readily extracted using THF at reflux. FT-IR spectroscopy studies confirmed the complete removal of the polystyrene chains (see Figure S1), and the density of the resulting broken silica shells was determined to be 1.93 g cm^–3^ at 20 °C by helium pycnometry. Accordingly, this value was used in conjunction with eq 1 to calculate the mean silica thicknesses summarized in Table 1.

The importance of appropriate surface modification using a cationic polymer such as chitosan is highlighted by a control experiment in which the bare 6 μm PMMA latex is utilized instead of the chitosan-coated particles. The SEM images shown in Figure 3a,c,e indicate extensive secondary nucleation of relatively large silica particles, with minimal surface deposition being achieved and no free-standing shells remaining after calcination. In striking contrast, silica deposition experiments conducted using chitosan-coated PMMA latex particles produced well-defined uniform silica overlayers that formed free-standing shells after calcination (see Figure 3b,d,f). The latter observation confirms the contiguous nature of the silica coating. Moreover, SEM analysis reveals the presence of surface cracks within the silica overlayers after drying silica-coated 6 μm PMMA latex particles (mean silica thickness = 144 nm) at 50 °C (data not shown). Presumably, this simply reflects the differing volumetric expansion coefficients for the PMMA and silica components. However, drying the same silica-coated latex more slowly at ambient temperature also resulted in surface cracks within the silica overlayers.

In principle, calcination is likely to lead to some degree of densification of the original silica overlayer. Nevertheless, the target silica shell appears to be in reasonably good agreement with the mean silica overlayer thickness calculated from the corresponding SEM image (see Figure S5). Hence this should provide independent validation of the various assumptions involved in the use of eq 1 (see above). In principle, this provides independent validation of the various assumptions involved in the use of eq 1 (see above). In practice, the error in the mean thickness of such silica overlayers is estimated to be ±10%, which encompasses a relatively wide range of possible silica densities (see Figure S6). Thus the prudent conclusion is that such SEM measurements are certainly consistent with the expected silica overlayer thicknesses, but their limited accuracy precludes a more definitive interpretation.

A mean silica shell thickness of 150 nm was targeted for 6, 10, and 15 μm chitosan-coated latex particles (Table 1). These three syntheses were conducted using a constant latex surface area of 0.166 m^2^. Thus the solids concentration increased from 8.2% for the 6 μm latex up to 18.6% for the 15 μm PMMA particles. SEM analysis of the silica-coated 10 and 15 μm PMMA latex particles indicated high silica surface coverages but significantly rougher silica shells (Figure S7). After calcination to remove the underlying latex, free-standing silica shells were obtained.

**Figure 5:**
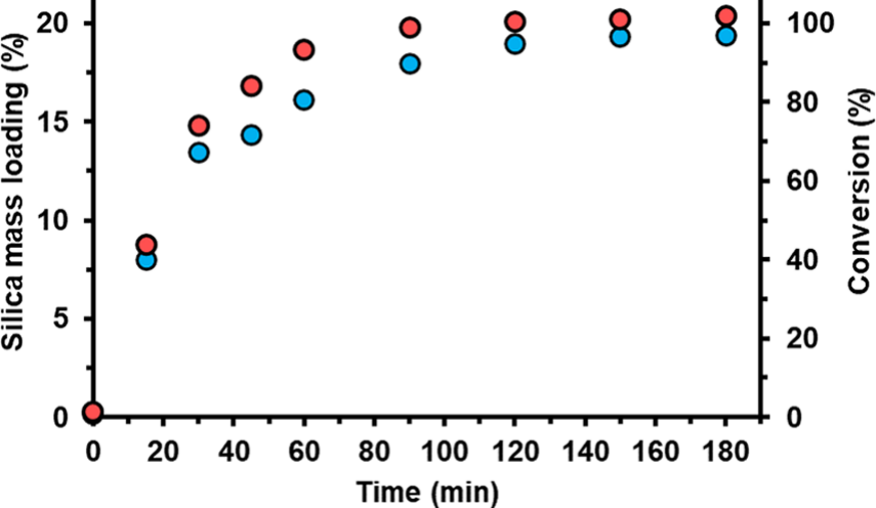
Conversion vs time curves obtained by thermogravimetry for Stöber silica formation in the presence of (a) bare 6 μm PMMA latex particles (red data set) and (b) chitosan-coated 6 μm PMMA latex particles (blue data set).

Thermogravimetry was also used to assess the kinetics of silica formation in the presence of the 6 μm PMMA latex particles. Approximately 95% conversion of TEOS was converted into silica within 2–3 h at 20 °C (see Figure 5). Interestingly, a slightly faster rate was observed in the absence of adsorbed chitosan chains. This suggests that the presence of this cationic biopolymer merely promotes surface deposition of the soluble silica precursor species and/or the adsorption of nascent silica nuclei (<5 nm diameter)^86^ rather than catalyzing silica formation.

**Figure 6:**
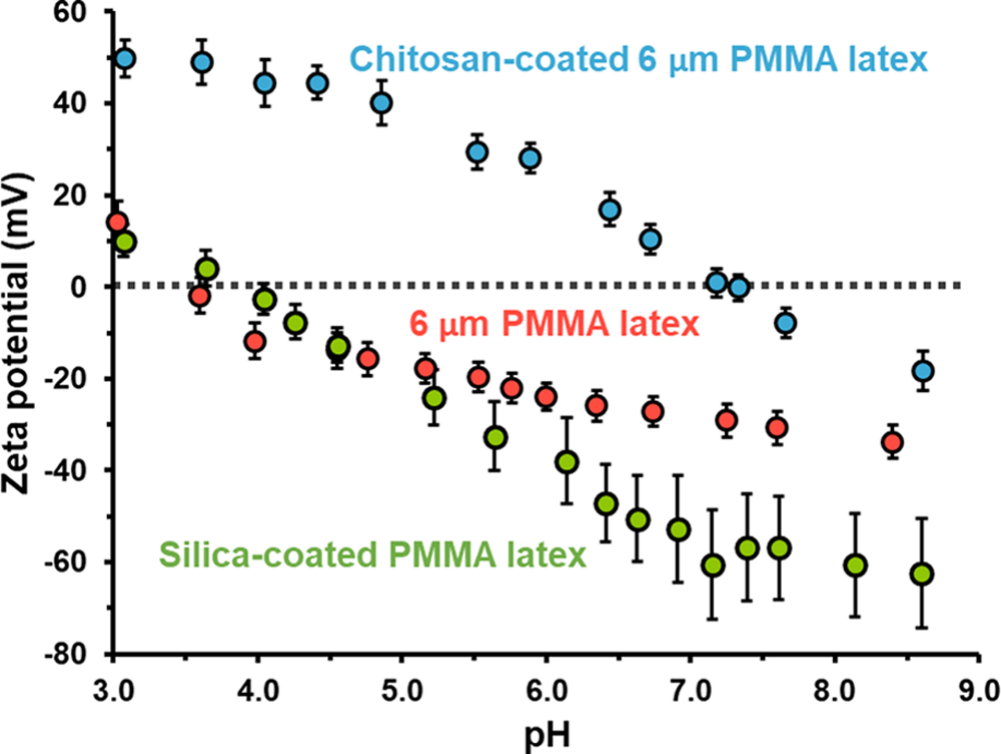
Zeta potential vs pH curves recorded for bare 6 μm PMMA latex, chitosan-coated 6 μm PMMA latex, and silica-coated 6 μm PMMA latex (prepared under optimized conditions in the presence of chitosan when targeting a silica overlayer thickness of 148 nm).

Zeta potential vs pH curves constructed for the bare 6 μm PMMA latex, a chitosan-coated 6 μm PMMA latex, and a silica-coated 6 μm PMMA latex (target silica overlayer thickness = 148 nm) are shown in Figure 6. The bare latex exhibits cationic character at pH 3, an isoelectric point (IEP) at around pH 3.50, and anionic character at pH 4 or above (with a limiting zeta potential of around −30 mV). In contrast, the IEP observed for the corresponding chitosan-coated PMMA latex is shifted to approximately pH 7.25, which is consistent with the surface presence of this primary amine-functional biopolymer. Finally, the silica-coated latex has an IEP of around 3.75, and its limiting zeta potential at high pH is around −60 mV. This is consistent with the formation of a contiguous silica shell surrounding each latex particle.

X-ray photoelectron survey spectra recorded for the bare 6 μm PMMA latex, chitosan alone, and a chitosan-coated 6 μm PMMA latex are shown in Figure S8. In principle, the bare latex particles should contain no surface nitrogen atoms, which would facilitate determination of the chitosan surface coverage. In practice, a N1s signal (4.3 at %) is observed, which suggests that a nitrogen-based polymer or surfactant was employed for the synthesis of these commercially sourced latex particles. Given that these latex particles are anionic at low pH, the nitrogen species on the latex surface do not appear to be an amine. Chitosan adsorption leads to only a modest increase in this N1s signal intensity, which makes accurate quantification of the surface coverage of this component somewhat problematic. Nevertheless, the surface charge reversal observed for the aqueous electrophoresis curves shown in Figure 6 provides strong evidence for chitosan-coated PMMA particles. Moreover, the presence of adsorbed chitosan chains clearly has a marked influence on the uniformity of the deposited silica overlayer (see Figure 3).

**Figure 7:**
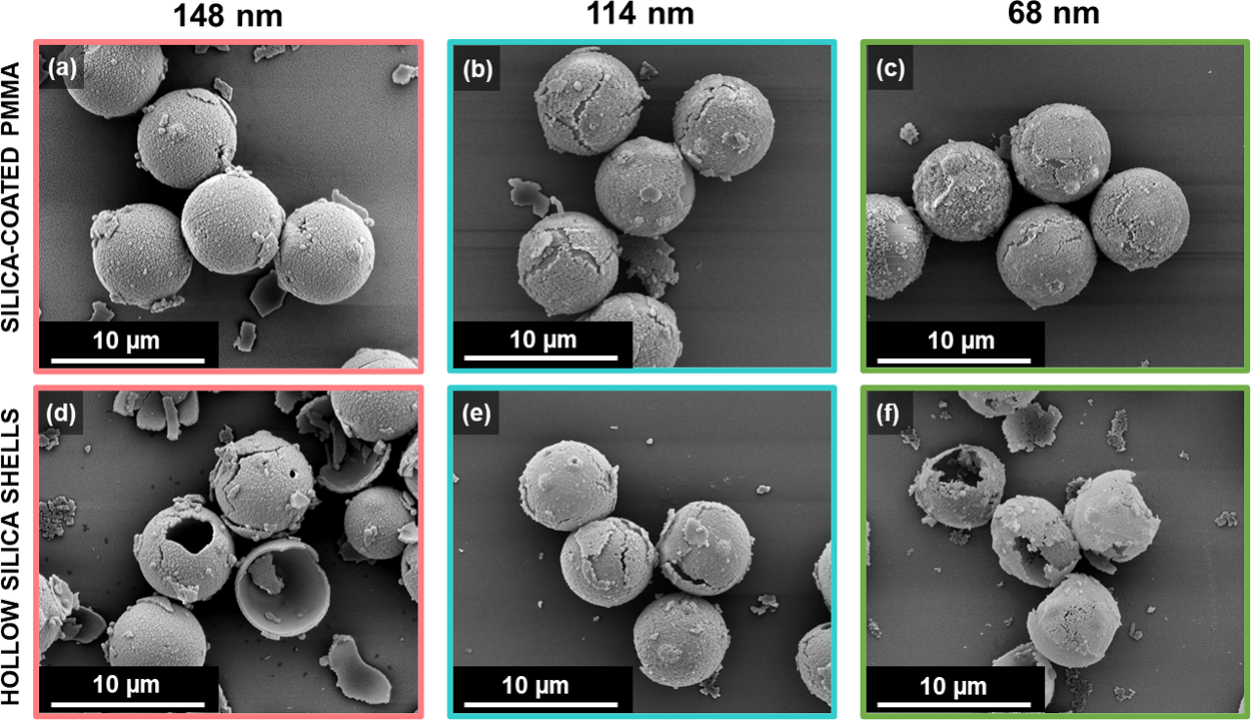
SEM images recorded for silica-coated 6 μm PMMA latex particles when targeting a mean silica overlayer thickness of (a) 148 nm, (b) 114 nm, or (c) 68 nm. SEM images recorded for the corresponding (d–f) hollow silica shells obtained after the calcination of such silica-coated latexes.

Survey spectra were also recorded for silica formed in the absence of any latex particles and for three silica-coated 6 μm PMMA latexes (target silica overlayer thicknesses = 68, 114, and 148 nm, respectively) as shown in Figure S9. Stöber-synthesized silica invariably contains a minor fraction of carbon atoms owing to unreacted ethoxy groups, which complicates calculation of the silica surface coverage via obscuration of the C1s signal attributed to the underlying PMMA latex. In view of this problem (and given that no Si2p signal was observed for the bare PMMA latex), we chose to compare the Si2p signal observed for silica-coated PMMA latex particles to that of silica alone. This approach enables surface coverages of 71–81% to be estimated for target silica thicknesses of 68–148 nm (see Table 2). XPS has a typical sampling depth of 2–10 nm,^87^ which is much less than the lowest silica overlayer thickness targeted in this study. Thus, essentially full surface coverage had been anticipated. However, these apparently incomplete coverages are almost certainly underestimated because SEM analysis revealed that extensive surface cracking of the brittle silica shells occurs on drying, which exposes the underlying PMMA latex to the incident X-ray beam (see Figure 7).

**Table 2:** XPS Si2p Spectra Recorded for Silica Formed in the Absence of Latex Particles but in the Presence of Chitosan and Silica-Coated 6 μm PMMA Latexes (Targeting 148, 114, and 68 nm Shell Thicknesses)^a^

sample	Si2p (%)	surface silica coverage (%)
silica control	31.11	100
148 nm silica shell	25.14	81
114 nm silica shell	24.20	78
68 nm silica shell	21.97	71

a Final surface silica coverage is assessed by comparing the silica content with that of the control silica sample.

## Conclusions

We report an efficient process-intensive protocol for the preparation of silica-coated micrometer-sized PMMA latex particles using a Stöber-type formulation when employing TEOS as a soluble silica precursor. Unlike many literature reports, this enables well-defined core-shell particles to be obtained at relatively high solids (8.2 to 23.5% w/w). The key to ensuring efficient silica deposition at the latex surface is the prior adsorption of chitosan. In the absence of this cationic biopolymer, silica deposition is poorly controlled and involves substantial secondary nucleation, as judged by SEM and optical microscopy studies. In contrast, the presence of chitosan promotes the surface deposition of a relatively uniform silica overlayer and minimizes the problem of secondary nucleation. Aqueous electrophoresis studies confirmed that the original latex particles are anionic at neutral pH, while the chitosan-coated latex particles are cationic and the silica-coated latex particles are anionic.

Kinetic studies indicated that silica deposition was complete within 2–3 h at 20 °C. No significant difference was observed in the presence or absence of adsorbed chitosan chains, so this component merely promotes silica deposition rather than acting as a catalyst. Silica mass contents were readily determined by thermogravimetry, and mean silica shell thicknesses were estimated by SEM studies. These data indicated that essentially all the TEOS is converted into silica and almost all of the silica is deposited onto the latex particles. Thus, for a given latex diameter, the mean silica shell thickness simply depends on the initial TEOS/latex mass ratio. Calcination of the silica-coated latex particles leads to the formation of well-defined silica shells, which suggests that the original silica overlayer was contiguous. Finally, XPS studies of three silica-coated latexes enabled silica surface coverages of 71–81% to be estimated by comparing their Si2p signal intensities to that of silica alone. However, such values are almost certainly an underestimation because the silica shells undergo extensive cracking on drying, which partially exposes the underlying PMMA latex cores.

## Supporting Information Available

Full experimental details and density measurements for hollow silica shells obtained using 20 μm linear polystyrene latex; FT-IR spectra and SEM images recorded for silica-coated polystyrene particles; additional laser diffraction data for chitosan-coated PMMA latex; optical microscopy images of bare and chitosan-coated PMMA latex; silica overlayer thickness vs silica density plot; SEM images recorded for silica-coated PMMA latexes of 6, 10, and 15 μm diameter; XPS survey spectra recorded for chitosan alone, bare PMMA latex, chitosan-coated PMMA latex, and silica-coated PMMA latexes when targeting various silica shell thicknesses (PDF)

## References

[ref1] Ottewill R. H.; Schofield A. B.; Waters J. A.; Williams N. S. J. Preparation of core-shell polymer colloid particles by encapsulation. Colloid Polym. Sci. 1997, 275, 274–283. 10.1007/s003960050081.

[ref2] Khan A. K.; Ray B. C.; Dolui S. K. Preparation of core-shell emulsion polymer and optimization of shell composition with respect to opacity of paint film. Prog. Org. Coat. 2008, 62, 65–70. 10.1016/j.porgcoat.2007.09.022.

[ref3] Kang E.; Graczykowski B.; Jonas U.; Christie D.; Gray L. A. G.; Cangialosi D.; Priestley R. D.; Fytas G. Shell architecture strongly influences the glass transition, surface mobility, and elasticity of polymer core-shell nanoparticles. Macromolecules 2019, 52, 5399–5406. 10.1021/acs.macromol.9b00766. 31367064 PMC6659035

[ref4] Bertuoli P. T.; Baldissera A. F.; Zattera A. J.; Ferreira C. A.; Alemán C.; Armelin E. Polyaniline coated core-shell polyacrylates: control of film formation and coating application for corrosion protection. Prog. Org. Coat. 2019, 128, 40–51. 10.1016/j.porgcoat.2018.12.007.

[ref5] Goulis P.; Kartsonakis I. A.; Charitidis C. A. Synthesis and characterization of a core-shell copolymer with different glass transition temperatures. Fibers 2020, 8, 71 10.3390/fib8110071.

[ref6] Lascelles S. F.; Armes S. P. Synthesis and characterization of micrometre-sized, polypyrrole-coated polystyrene latexes. J. Mater. Chem. 1997, 7, 1339–1347. 10.1039/a700237h.

[ref7] Khan M. A.; Armes S. P. Synthesis and characterization of micrometer-sized poly(3,4-ethylenedioxythiophene)-coated polystyrene latexes. Langmuir 1999, 15, 3469–3475. 10.1021/la9815897.

[ref8] Khan M. A.; Armes S. P.; Perruchot C.; Ouamara H.; Chehimi M. M.; Greaves S. J.; Watts J. F. Surface characterization of poly(3,4-ethylenedioxythiophene)-coated latexes by x-ray photoelectron spectroscopy. Langmuir 2000, 16, 4171–4179. 10.1021/la991390+.

[ref9] Ormond-Prout J.; Dupin D.; Armes S. P.; Foster N. J.; Burchell M. J. Synthesis and characterization of polypyrrole-coated poly(methyl methacrylate) latex particles. J. Mater. Chem. 2009, 19, 1433–1442. 10.1039/b816839c.

[ref10] Fujii S.; Matsuzawa S.; Nakamura Y. One-pot synthesis of conducting polymer -coated latex particles: ammonium persulfate as free radical initiator and chemical oxidant. Chem. Commun. 2010, 46, 7217–7219. 10.1039/c0cc02005b. 20820522

[ref11] Goldsworthy B. J.; Burchell M. J.; Cole M. J.; Armes S. P.; Khan M. A.; Lascelles S. F.; Green S. F.; McDonnell J. A. M.; Srama R.; Bigger S. W. Time of flight mass spectra of ions in plasmas produced by hypervelocity impacts of organic and mineralogical microparticles on a cosmic dust analyser. Astron. Astrophys. 2003, 409, 1151–1167. 10.1051/0004-6361:20031087.

[ref12] Burchell M. J.; Foster N. J.; Ormond-Prout J.; Dupin D.; Armes S. P. Extent of thermal ablation suffered by model organic microparticles during aerogel capture at hypervelocities. Meteorit. Planet. Sci. 2009, 44, 1407–1419.

[ref13] Fujii S.; Matsuzawa S.; Nakamura Y.; Ohtaka A.; Teratani T.; Akamatsu K.; Tsuruoka T.; Nawafune H. Synthesis and characterization of polypyrrole-palladium nanocomposite-coated latex particles and their use as a catalyst for Suzuki coupling reaction in aqueous media. Langmuir 2010, 26, 6230–6239. 10.1021/la9039545. 20146495

[ref14] Fujii S.; Matsuzawa S.; Hamasaki H.; Nakamura Y.; Bouleghlimat A.; Buurma N. J. Polypyrrole-palladium nanocomposite coating of micrometer-sized polymer particles toward a recyclable catalyst. Langmuir 2012, 28, 2436–2447. 10.1021/la204324f. 22204384

[ref15] Tierno P.; Goedel W. A. Using electroless deposition for the preparation of micron sized polymer/metal core/shell particles and hollow metal spheres. J. Phys. Chem. B 2006, 110, 3043–3050. 10.1021/jp054213s. 16494306

[ref16] Strohm H.; Löbmann P. Liquid-phase deposition of tio2 on polystyrene latex particles functionalized by the adsorption of polyelectrolytes. Chem. Mater. 2005, 17, 6772–6780. 10.1021/cm051345p.

[ref17] Yuan J.-J.; Mykhaylyk O. O.; Ryan A. J.; Armes S. P. Cross-linking of cationic block copolymer micelles by silica deposition. J. Am. Chem. Soc. 2007, 129, 1717–1723. 10.1021/ja0674946. 17249673

[ref18] Zou H.; Wu S.; Shen J. Polymer/silica nanocomposites: preparation, characterization, properties, and applications. Chem. Rev. 2008, 108, 3893–3957. 10.1021/cr068035q. 18720998

[ref19] Balmer J. A.; Schmid A.; Armes S. P. Colloidal nanocomposite particles: quo vadis?. J. Mater. Chem. 2008, 18, 5722–5730. 10.1039/b805764h.

[ref20] Zou H.; Schlaad H. Sodium silicate route to coat polymer particles with silica. Colloid Polym. Sci. 2014, 292, 1693–1700. 10.1007/s00396-014-3229-5.

[ref21] Bechert B.; Hümmer W.; Schwatz M.; Wiese H. Aqueous compositions comprising a film forming polymer and an anionic emulsifier, BASF, EP0982279B2, 2000.

[ref22] Hwang H. S.; Lee S. B.; Park I. Fabrication of raspberry-like superhydrophobic hollow silica particles. Mater. Lett. 2010, 64, 2159–2162. 10.1016/j.matlet.2010.07.031.

[ref23] Gong X.; He S. Highly durable superhydrophobic polydimethylsiloxane/silica nanocomposite surfaces with good self-cleaning ability. ACS Omega 2020, 5, 4100–4108. 10.1021/acsomega.9b03775. 32149238 PMC7057699

[ref24] Arfsten N. J.; Armes S. P.; Buskens P. J. P.; Thies J. C.; Vrijaldenhoven P. W. A. Coating composition comprising core-shell nanoparticles, DSM Research, EP 2059836 B1, 2007.

[ref25] Zhang X.; Lan P.; Lu Y.; Li J.; Xu H.; Zhang J.; Lee Y.; Rhee J. Y.; Choy K.-L.; Song W. Multifunctional antireflection coatings based on novel hollow silica–silica nanocomposites. ACS Appl. Mater. Interfaces 2014, 6, 1415–1423. 10.1021/am405258d. 24443948

[ref26] Retsch M.; Schmelzeisen M.; Butt H.-J.; Thomas E. L. Visible Mie scattering in nonabsorbing hollow sphere powders. Nano Lett. 2011, 11, 1389–1394. 10.1021/nl2002445. 21338055

[ref27] Fielding L. A.; Mykhaylyk O. O.; Schmid A.; Pontoni D.; Armes S. P.; Fowler P. W. Visible Mie scattering from hollow silica particles with particulate shells. Chem. Mater. 2014, 26, 1270–1277. 10.1021/cm4039347.

[ref28] Graf C.; Vossen D. L. J.; Imhof A.; van Blaaderen A. A general method to coat colloidal particles with silica. Langmuir 2003, 19, 6693–6700. 10.1021/la0347859. 20334440

[ref29] Grady Z. A.; Arthur A. Z.; Wohl C. J. Topological control of polystyrene-silica core-shell microspheres. Colloids Surf., A 2019, 560, 136–140. 10.1016/j.colsurfa.2018.10.019. PMC7816762 33479556

[ref30] Balmer J. A.; Armes S. P.; Fowler P. W.; Tarnai T.; Asp Ar Z. G.; Murray K. A.; Williams N. S. J. Packing efficiency of small silica particles on large latex particles: a facile route to colloidal nanocomposites. Langmuir 2009, 25, 5339–5347. 10.1021/la8041555. 19260684

[ref31] Abdollahi H.; Ershad-Langroudi A.; Salimi A.; Rahimi A. Anticorrosive coatings prepared using epoxy-silica hybrid nanocomposite materials. Ind. Eng. Chem. Res. 2014, 53, 10858–10869. 10.1021/ie501289g.

[ref32] Kim T. H.; Song K. C. Low-temperature preparation of superhydrophilic coatings using tetraethoxysilane and colloidal silica by sol-gel method. Colloids Surf., A 2022, 647, 129105 10.1016/j.colsurfa.2022.129105.

[ref33] Calderon-Guillen J. A.; Aviles-Arellano L. M.; Pérez-Robles J. F.; Gonzalez-Hernández J.; Ramos-Ramírez E. Dense silica-based coatings prepared from colloidal silica. Surf. Coat. Technol. 2005, 190, 110–114. 10.1016/j.surfcoat.2004.04.068.

[ref34] Yang Y.; Li F.; Xiao M.; Zhang Z.; Wei J.; Hu J.; Yu Q. TEOS and Na_2_SiO_3_ as silica sources: study of synthesis and characterization of hollow silica nanospheres as nano thermal insulation materials. Appl. Nanosci. 2020, 10, 1833–1844. 10.1007/s13204-020-01330-0.

[ref35] El-Nahhal I. M.; Salem J. K.; Kuhn S.; Hammad T.; Hempelmann R.; al Bhaisi S. Synthesis and characterization of silica coated and functionalized silica coated zinc oxide nanomaterials. Powder Technol. 2016, 287, 439–446. 10.1016/j.powtec.2015.09.042.

[ref36] Zou H.; Wu S.; Shen J. Preparation of silica-coated poly(styrene-co-4-vinylpyridine) particles and hollow particles. Langmuir 2008, 24, 10453–10461. 10.1021/la800366j. 18698854

[ref37] Zhang L.; D’Acunzi M.; Kappl M.; Auernhammer G. K.; Vollmer D.; van Kats C. M.; van Blaaderen A. Hollow silica spheres: synthesis and mechanical properties. Langmuir 2009, 25, 2711–2717. 10.1021/la803546r. 19437752

[ref38] Gorsd M. N.; Pizzio L. R.; Blanco M. N. Synthesis and characterization of hollow silica spheres. Proc. Mater. Sci. 2015, 8, 567–576. 10.1016/j.mspro.2015.04.110.

[ref39] Ding X.; Yu K.; Jiang Y.; Hari-Bala; Zhang H.; Wang Z. A novel approach to the synthesis of hollow silica nanoparticles. Mater. Lett. 2004, 58, 3618–3621.

[ref40] Sun B.; Mutch S. A.; Lorenz R. M.; Chiu D. T. Layered polyelectrolyte-silica coating for nanocapsules. Langmuir 2005, 21, 10763–10769. 10.1021/la0513297. 16262349

[ref41] Tan B.; Rankin S. E. Dual latex/surfactant templating of hollow spherical silica particles with ordered mesoporous shells. Langmuir 2005, 21, 8180–8187. 10.1021/la050618s. 16114920

[ref42] Whitaker K. A.; Furst E. M. Layer-by-layer synthesis of mechanically robust solvent-permeable silica nanoshells. Langmuir 2014, 30, 584–591. 10.1021/la402737f. 24443919

[ref43] Hu P.; Ai D.; Jiang X.; Zhang X. Fabrication of hollow silica nanosphere and its application for thermal insulation coating. J. Thermoplast. Compos. Mater. 2020, 33, 198–213. 10.1177/0892705718805133.

[ref44] Tissot I.; Reymond J. P.; Lefebvre F.; Bourgeat-Lami E. SiOH-functionalized polystyrene latexes. a step toward the synthesis of hollow silica nanoparticles. Chem. Mater. 2002, 14, 1325–1331. 10.1021/cm0112441.

[ref45] Cho B. Preparation of hollow porous silica nanospheres and their potential for glucagon-like peptide-1 delivery. Mater. Res. Express 2019, 6, 045016 10.1088/2053-1591/aafb3d.

[ref46] Chen Y.; Kang E. T.; Neoh K. G.; Greiner A. Preparation of hollow silica nanospheres by surface-initiated atom transfer radical polymerization on polymer latex templates. Adv. Funct. Mater. 2005, 15, 113–117. 10.1002/adfm.200400179.

[ref47] Zhang F.; Sun J.; Shiyang B.; Wu X. Synthesis and characterization of hollow mesoporous silica spheres with tunable shell thicknesses and its application in ibuprofen delivery. J. Porous Mater. 2018, 25, 581–593. 10.1007/s10934-017-0471-5.

[ref48] Zhou S. Z.; Qiao X. G. Synthesis of raspberry-like polymer@silica hybrid colloidal particles through biphasic sol-gel process. Colloids Surf., A 2018, 553, 230–236. 10.1016/j.colsurfa.2018.05.040.

[ref49] Chen M.; Zhou S.; Wu L.; Xie S.; Chen Y. Preparation of silica-coated polystyrene hybrid spherical colloids. Macromol. Chem. Phys. 2005, 206, 1896–1902. 10.1002/macp.200500200.

[ref50] Kim K. D.; Kim H. T. Formation of silica nanoparticles by hydrolysis of TEOS using a mixed semi-batch/batch method. J. Sol-Gel Sci. Technol. 2002, 25, 183–189. 10.1023/A:1020217105290.

[ref51] Ge C.; Zhang D.; Wang A.; Yin H.; Ren M.; Liu Y.; Jiang T.; Yu L. Synthesis of porous hollow silica spheres using polystyrene-methyl acrylic acid latex template at different temperatures. J. Phys. Chem. Solids 2009, 70, 1432–1437. 10.1016/j.jpcs.2009.08.013.

[ref52] Zhou H.; Luo J.; Gao Q.; Yang T. Latex-templated biomineralization of silica nanoparticles with porous shell and their application for drug delivery. J. Appl. Polym. Sci. 2016, 133, 44200.

[ref53] Xu S. W.; Lu Y.; Li J.; Zhang Y. F.; Jiang Z. Y. Preparation of novel silica-coated alginate gel beads for efficient encapsulation of yeast alcohol dehydrogenase. J. Biomater. Sci., Polym. Ed. 2007, 18, 71–80. 10.1163/156856207779146141. 17274452

[ref54] Latthe S. S.; Nadargi D. Y.; Venkateswara Rao A. TMOS based water repellent silica thin films by co-precursor method using TMES as a hydrophobic agent. Appl. Surf. Sci. 2009, 255, 3600–3604. 10.1016/j.apsusc.2008.10.005.

[ref55] Xu H.; Yan F.; Monson E. E.; Kopelman R. Room-temperature preparation and characterization of poly (ethylene glycol)-coated silica nanoparticles for biomedical applications. J. Biomed. Mater. Res. Part A 2003, 66A, 870–879. 10.1002/jbm.a.10057. 12926040

[ref56] Yang J.; Lind J. U.; Trogler W. C. Synthesis of hollow silica and titania nanospheres. Chem. Mater. 2008, 20, 2875–2877. 10.1021/cm703264y.

[ref57] Iler R. K. Product comprising a skin of dense, hydrated amorphous silica bound upon a core of another solid material and the process of making same, E.I. du Pont de Nemours and Co., US 2885366 A, 1959.

[ref58] Cornelissen J. J. L. M.; Connor E. F.; Kim H.-C.; Lee V. Y.; Magibitang T.; Rice P. M.; Volksen W.; Sundberg L. K.; Miller R. D. Versatile synthesis of nanometer sized hollow silica spheres. Chem. Commun. 2003, 1010–1011. 10.1039/b212811j. 12744344

[ref59] Lee C.; Kim J.; Chang H.; Roh K.-M.; Dong Jang H. Synthesis of hollow silica particles from sodium silicate using organic template particles. Kor. Chem. Eng. Res. 2015, 53, 78–82. 10.9713/kcer.2015.53.1.78.

[ref60] Liu C.; Ge C.; Wang A.; Yin H.; Ren M.; Zhang Y.; Yu L.; Jiang T. Synthesis of porous hollow silica spheres using functionalized polystyrene latex spheres as templates. Korean J. Chem. Eng. 2011, 28, 1458–1463. 10.1007/s11814-010-0366-5.

[ref61] Manning J. R. H.; Walkley B.; Provis J. L.; Patwardhan S. Mimicking biosintering: the identification of highly condensed surfaces in bioinspired silica materials. Langmuir 2021, 37, 561–568. 10.1021/acs.langmuir.0c03261. 33372796 PMC7815198

[ref62] Iler R. K. The Chemistry of Silica: Solubility, Polymerization, Colloid and Surface Properties and Biochemistry*.*; Wiley: New York, 1979.

[ref63] Coradin T.; Roux C.; Livage J. Biomimetic self-activated formation of multi-scale porous silica in the presence of arginine-based surfactants. J. Mater. Chem. 2002, 12, 1242–1244. 10.1039/b201616h.

[ref64] Brinker C. J.; Scherer G. W. Sol-Gel Science: The Physics and Chemistry of Sol-Gel Processing, 1st ed.; Academic Press: San Diego, 1990.

[ref65] Stöber W.; Fink A.; Bohn E. Controlled growth of monodisperse silica spheres in the micron size range. J. Colloid Interface Sci. 1968, 26, 62–69. 10.1016/0021-9797(68)90272-5.

[ref66] Issa A. A.; Luyt A. S. Kinetics of alkoxysilanes and organoalkoxysilanes polymerization: a review. Polymers 2019, 11, 537 10.3390/polym11030537. 30960521 PMC6473841

[ref67] Hyde E. D. E. R.; Seyfaee A.; Neville F.; Moreno-Atanasio R. Colloidal silica particle synthesis and future industrial manufacturing pathways: a review. Ind. Eng. Chem. Res. 2016, 55, 8891–8913. 10.1021/acs.iecr.6b01839.

[ref68] Li S.; Wang J.; Qu W.; Cheng J.; Lei Y.; Liu M.; Wang D. Epoxy/nano-sio2 anticorrosion coatings synthesized by different molar ratio of tetraethyl orthosilicate (TEOS) and tetramethyl orthosilicate (TMOS). Int. J. Electrochem. Sci. 2019, 14, 11641–11650.

[ref69] Wu G.; An J.; Sun D.; Tang X.; Xiang Y.; Yang J. Robust microcapsules with polyurea/silica hybrid shell for one-part self-healing anticorrosion coatings. J. Mater. Chem. A 2014, 2, 11614–11620. 10.1039/C4TA01312C.

[ref70] Barrena M. I.; de Salazar J. M. G.; Soria A.; Matesanz L. Pre-hydrolysed ethyl silicate as an alternative precursor for SiO_2_-coated carbon nanofibers. Appl. Surf. Sci. 2011, 258, 1212–1216. 10.1016/j.apsusc.2011.09.073.

[ref71] Niu L.; Xia Z.; Lei L.; Zhang Y.; Zhong L. Sol-gel process of alkoxysilane in emulsifier-involved aqueous emulsions: a one-pot synthetic route to emulsions of core-shell composite particles and their applications. J. Appl. Polym. Sci. 2013, 128, 4237–4244. 10.1002/app.38660.

[ref72] Antonietti M.; Berton B.; Göltner C.; Hentze H.-P. Synthesis of mesoporous silica with large pores and bimodal pore size distribution by templating of polymer latices. Adv. Mater. 1998, 10, 154–159. 10.1002/(SICI)1521-4095(199801)10:2<154::AID-ADMA154>3.0.CO;2-I.

[ref73] Liz-Marzán L. M.; Giersig M.; Mulvaney P. Synthesis of nanosized gold–silica core–shell particles; synthesis of nanosized gold–silica core–shell particles. Langmuir 1996, 12, 4329–4335. 10.1021/la9601871.

[ref74] Zhou F.; Li S.; Vo C. D.; Yuan J. J.; Chai S.; Gao Q.; Armes S. P.; Lu C.; Cheng S. Biomimetic deposition of silica templated by a cationic polyamine-containing microgel. Langmuir 2007, 23, 9737–9744. 10.1021/la700715t. 17685562

[ref75] Yun D. S.; Jang H. G.; Yoo J. W. Fabrication of uniform hollow silica nanospheres using a cationic polystyrene core. Bull. Korean Chem. Soc. 2011, 32, 1534–1538. 10.5012/bkcs.2011.32.5.1534.

[ref76] Jenjob S.; Sunintaboon P.; Inprakhon P.; Anantachoke N.; Reutrakul V. Chitosan-functionalized poly(methyl methacrylate) particles by spinning disk processing for lipase immobilization. Carbohydr. Polym. 2012, 89, 842–848. 10.1016/j.carbpol.2012.04.019. 24750870

[ref77] Budnyak T. M.; Pylypchuk I. V.; Tertykh V. A.; Yanovska E. S.; Kolodynska D. Synthesis and adsorption properties of chitosan-silica nanocomposite prepared by sol-gel method. Nanoscale Res. Lett. 2015, 10, 1–10. 25852383 10.1186/s11671-014-0722-1 PMC4385279

[ref78] Fleer G. J.; Cohen Stuart M. A.; Scheutjens J. M. H. M.; Cosgrove T.; Vincent B. Polymers at Interfaces; Chapman and Hall: London, UK, 1993.

[ref79] Williams M.; Armes S. P.; York D. W. Clay-based colloidosomes. Langmuir 2012, 28, 1142–1148. 10.1021/la2046405. 22145757

[ref80] Kashiwagi T.; Brown J. E.; Inaba A.; Hatada K.; Kitayama T.; Masuda E. Effects of weak linkages on the thermal and oxidative degradation of poly(methyl methacrylates). Macromolecules 1986, 19, 2160–2168. 10.1021/ma00162a010.

[ref81] Giesche H. Fine Particles: Synthesis, Characterization, and Mechanisms of Growth: Surfactant Series, 1st ed.; Tadao, Sugimoto, Ed.; CRC Press: Boca Raton, 2000; Vol. 92.

[ref82] Plumeré N.; Ruff A.; Speiser B.; Feldmann V.; Mayer H. A. Stöber silica particles as basis for redox modifications: particle shape, size, polydispersity, and porosity. J. Colloid Interface Sci. 2012, 368, 208–219. 10.1016/j.jcis.2011.10.070. 22169182

[ref83] Parnell S. R.; Washington A. L.; Parnell A. J.; Walsh A.; Dalgliesh R. M.; Li F.; Hamilton W. A.; Prevost S.; Fairclough J. P. A.; Pynn R. Porosity of silica Stöber particles determined by spin-echo small angle neutron scattering. Soft Matter 2016, 12, 4709–4714. 10.1039/C5SM02772A. 27021920

[ref84] van Blaaderen A.; Vrij A. Synthesis and characterization of monodisperse colloidal organo-silica spheres. J. Colloid Interface Sci. 1993, 156, 1–18. 10.1006/jcis.1993.1073.

[ref85] van Blaaderen A.; Kentgens A. P. M. Particle morphology and chemical microstructure of colloidal silica spheres made from alkoxysilanes. J. Non-Cryst. Solids 1992, 149, 161–178. 10.1016/0022-3093(92)90064-Q.

[ref86] Carcouët C. C. M. C.; van de Put M. W. P.; Mezari B.; Magusin P. C. M. M.; Laven J.; Bomans P. H. H.; Friedrich H.; Esteves A. C. C.; Sommerdijk N. A. J. M.; van Benthem R. A. T. M.; et al. Nucleation and growth of monodisperse silica nanoparticles. Nano Lett. 2014, 14, 1433–1438. 10.1021/nl404550d. 24499132

[ref87] Watts J. F.; Wolstenholme J. An Introduction to Surface Analysis by XPS and AES; Wiley: Chichester, 2003.

