# Supplementary material for: Silica-Coated Micrometer-Sized Latex Particles
Source: Langmuir. 2023 Mar 31;39(14):5169–78. doi: 10.1021/acs.langmuir.3c00227 (PMC10100546; doi:10.1021/acs.langmuir.3c00227)
Supplement: Supplementary file 1 — la3c00227_si_001.pdf [file la3c00227_si_001.pdf]

Supporting Information for

Silica-Coated Micron-Sized Latex Particles

O. Norvilaite[a], C. Lindsay[b], P. Taylor[b,] and S. P. Armes[a,*]

a. Dainton Building, Department of Chemistry, University of Sheffield, Brook Hill, Sheffield, South Yorkshire, S3 7HF, UK.

b. Syngenta, Jealott's Hill International Research Centre, Bracknell, Berkshire, RG42 6EY, UK.

Table of Contents Summary

Supporting Experiments and Analysis

Adsorption of chitosan onto linear 20 µm polystyrene latex particles

Silica deposition onto chitosan-coated 20 µm polystyrene latex particles

THF extraction of polystyrene cores to produce hollow silica shells

Silica density calculations

Supporting Table and Figures

Table S1. Summary of solid-state densities

Figure S1. FT-IR spectra recorded for silica-coated polystyrene latex particles before and after extraction of the polystyrene cores using THF

Figure S2. SEM images recorded for silica-coated polystyrene latex particles before and after extraction of the polystyrene cores using THF

Figure S3. Effect of order of addition for chitosan adsorption onto PMMA latex particles

Figure S4. Optical microscopy images for bare and chitosan-coated PMMA latex before silica deposition, after silica deposition, and after calcination

Figure S5. Silica shell thicknesses estimated from representative SEM images

Figure S6. Silica overlayer thickness vs. silica shell density calculation

Figure S7. SEM images recorded for silica-coated 6, 10 and 15 µm PMMA latex particles

Figure S8. XPS survey spectra for chitosan, bare PMMA latex and chitosan-coated PMMA latex

Figure S9. XPS survey spectra for three silica-coated PMMA latexes

Supporting Experiments and Analysis

Adsorption of chitosan onto 20 μm polystyrene latex particles

A 536 μL aliquot of a 0.20 g dm^{-3} aqueous solution of chitosan dissolved in 0.1 M acetic acid (corresponding to 1.071 mg chitosan) was diluted with deionized water (134.464 g). Dried linear 20 μm polystyrene latex (15.0 g) was added in approximately 2.0 g portions to this dilute acidic solution with vigorous mixing between each addition. The pH of the final latex suspension was 3.9. After magnetic stirring this acidic suspension for 16 h at 20 °C, the chitosan-coated polystyrene latex particles were isolated by freeze-drying overnight.

Silica deposition onto chitosan-coated 20 μm polystyrene latex particles

Chitosan-coated 20 μm polystyrene latex (10.044 g; total surface area = 1.434 m^2) was dispersed in a 10% v/v solution of TEOS in ethanol (40.882 mL, 18.308 mmol TEOS, target silica thickness = 202 nm) with the aid of magnetic stirring for 5 min. An aliquot of 28% ammonium hydroxide (8.994 mL) was added to this polystyrene latex suspension and the reaction mixture was stirred for 3 h at 20 °C. The resulting silica-coated PS latex particles were sedimented via centrifugation (5,000 rpm for 10 min; Beckman Coulter Avanti J-25 centrifuge), followed by redispersion first in ethanol (three times) and then in methanol (once). After decanting the final supernatant, the silica-coated PS latex particles were allowed to dry at 20 °C overnight.

THF extraction of polystyrene cores to produce hollow silica shells

To determine the solid-state density of the silica overlayer, the linear polystyrene chains were extracted using THF at reflux for 5 h. The remaining hollow silica shells were sedimented via centrifugation (5,000 rpm for 20 min), followed by redispersion in THF (five times) to ensure complete removal of the polystyrene. These hollow silica shells were then dried at 20 °C overnight. Successful extraction was confirmed using optical microscopy, SEM and FT-IR spectroscopy.

Silica density calculations

$$\boldsymbol{\rho_{particle}} = \frac{\boldsymbol{\rho_{core}V_{core} + \rho_{shell}V_{shell}}}{\boldsymbol{V_{particle}}} \quad \textbf{(S1)}$$

$$\boldsymbol{\rho_{particle}} = \frac{\boldsymbol{\rho_{core}R_{core}}^3 + \boldsymbol{\rho_{shell}}\left[(\boldsymbol{R_{core}} + \boldsymbol{T_{shell}})^3 - \boldsymbol{R_{core}}^3\right]}{(\boldsymbol{R_{core}} + \boldsymbol{T_{shell}})^3} \quad \textbf{(S2)}$$

$$\boldsymbol{\rho_{shell}} = \frac{\boldsymbol{\rho_{particle}}(\boldsymbol{R_{core}} + \boldsymbol{T_{shell}})^3 - \boldsymbol{\rho_{core}R_{core}}^3}{(\boldsymbol{R_{core}} + \boldsymbol{T_{shell}})^3 - \boldsymbol{R_{core}}^3} \quad \textbf{(S3)}$$

The density of core-shell particles ($\rho_{particle}$) can be calculated using **Equation S2**, which is derived from **Equation S1** (see B. Akpinar et al., *Macromolecules*, **2016**, *49*, 5160-5171). Rearranging **Equation S2** enables the density of the silica shell (ρ_{shell}) to be calculated using **Equation S3**.

The silica overlayer thickness was estimated from SEM images recorded for the silica-coated 20 μm polystyrene latex particles using ImageJ software. A mean silica shell thickness of 198 ± 22 nm was calculated by averaging thirty measurements. The densities of the silica-coated ($\rho_{particle}$) and chitosan-coated (ρ_{core}) particles are listed in **Table S1**. The particle radius (R_{core}) was taken to be 10,000 nm and the above shell thickness (T_{shell}) was used to estimate the density of the silica overlayer (1.9915 g cm^{-3}). This density is slightly greater than that determined by helium pycnometry (1.9296 g cm^{-3}). This is most likely because the calculated density does not account for the larger silica particles observed at surface of the silica overlayer.

Table S1. Summary of the density data obtained via helium pycnometry for chitosan-coated 20 μm polystyrene latex, silica-coated polystyrene latex particles and the hollow silica shells obtained after THF extraction of the linear polystyrene chains.

**Sample**	**Density (g cm^{-3})**	**Standard Deviation**
**Chitosan-coated polystyrene latex**	**1.074**	**0.0004**
**Silica-coated polystyrene latex**	**1.127**	**0.0004**
**Hollow silica shells**	**1.930**	**0.0023**

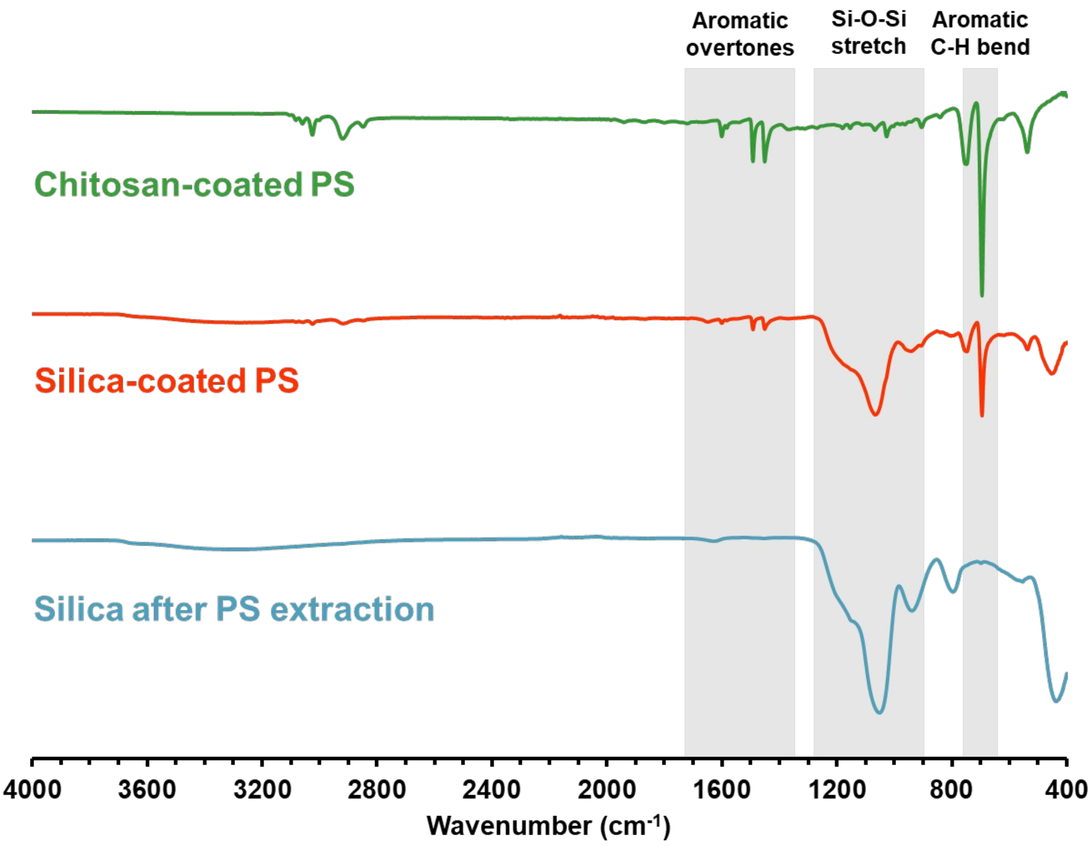

Figure S1. FT-IR spectra recorded for chitosan-coated polystyrene (PS) latex, silica-coated polystyrene latex and hollow silica shells obtained after extraction of linear polystyrene chains using THF at reflux. The absence of the aromatic overtones and C-H bend bands after such extraction (blue spectrum) indicates complete removal of the latex core.

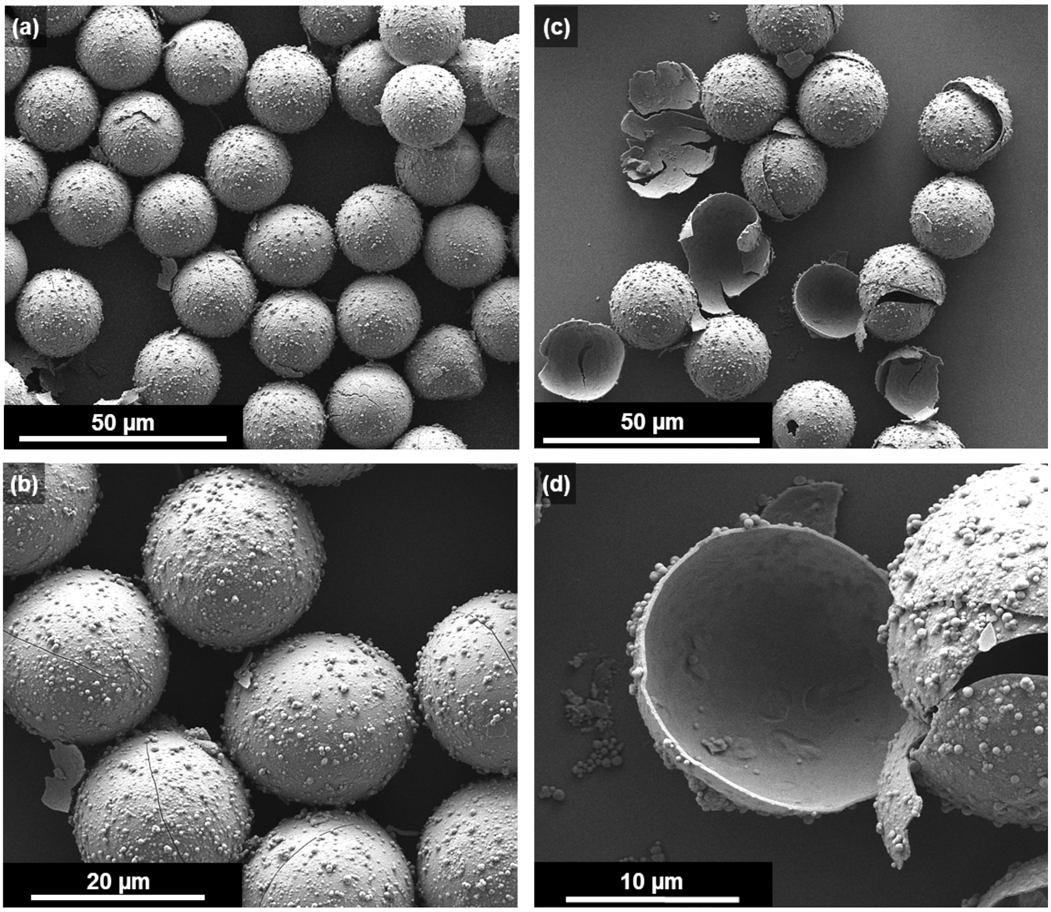

Figure S2. SEM images recorded for silica-coated 20 μm polystyrene latex particles (a-b) and the hollow silica shells obtained following extraction of linear polystyrene chains using THF at reflux (c-d).

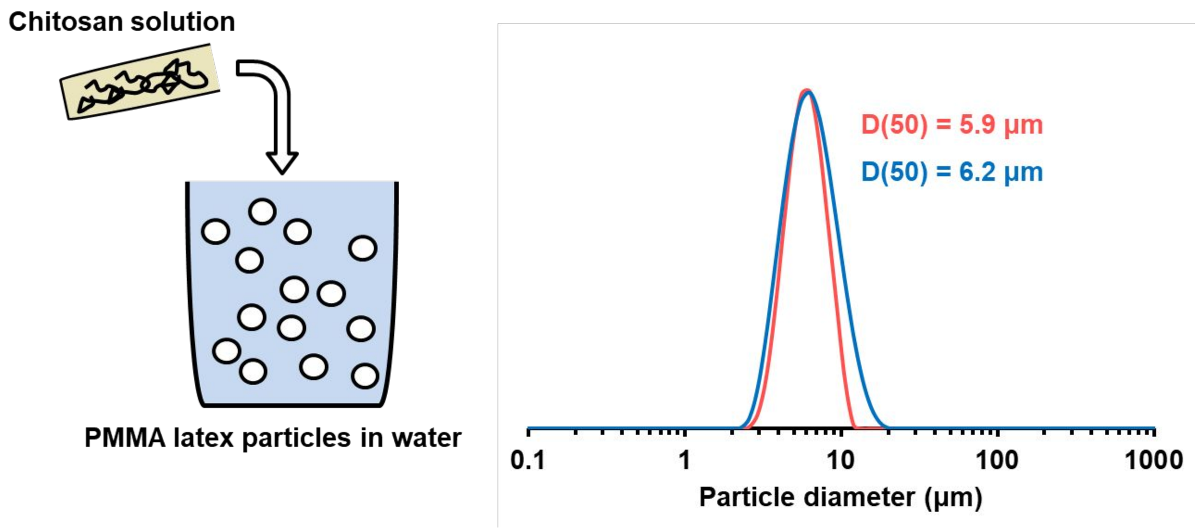

Figure S3. Laser diffraction particle size distributions recorded for PMMA latex particles before (red) and after (blue) adsorption of chitosan. In this set of experiments, the chitosan was added to the 6 µm PMMA latex particles, which leads to bridging flocculation.

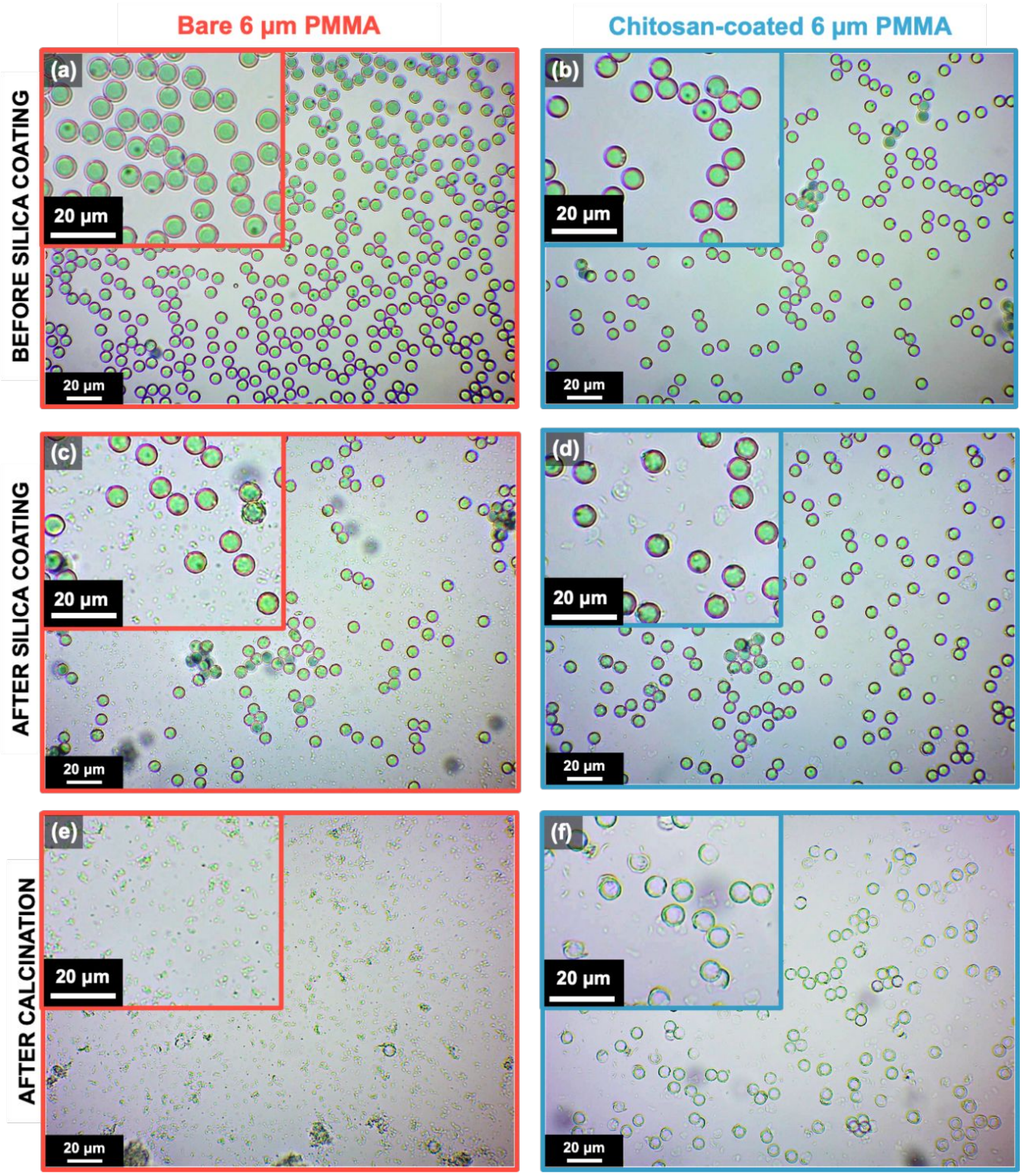

Figure S4. Optical microscopy images recorded for (a) the bare 6 μm PMMA latex, (b) chitosan-coated 6 μm PMMA latex, (c) attempted silica coating of PMMA latex in the absence of chitosan; (d) silica-coated PMMA latex prepared in the presence of chitosan; (e) attempted silica coating of PMMA latex in the absence of chitosan after calcination (no evidence for formation of hollow silica shells); (f) silica-coated PMMA latex prepared in the presence of chitosan after calcination (note formation of silica hollow shells).

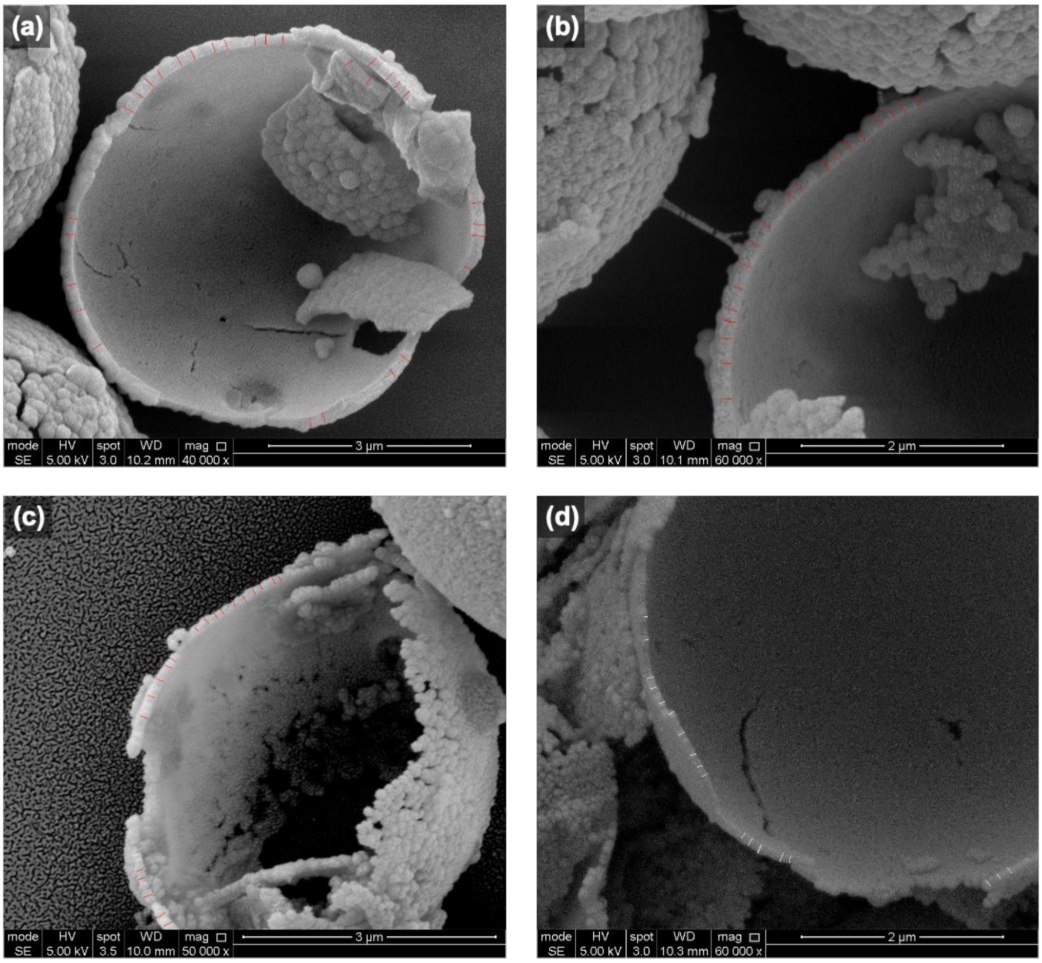

Image	Shell thickness calculated using Equation 1 (nm)	Shell thickness measured from SEM images (nm)
(a)	144	152 ± 23
(b)	112	131 ± 12
(c)	66	84 ± 14
(d)	45	66 ± 9

Figure S5. Representative SEM images of free-standing silica shells of varying shell thickness. ImageJ software was employed to analyse these (and other) images to estimate a mean silica shell thickness, with 20-30 measurements (see red or white lines) being made for at least two shells. Such silica shell thicknesses are in reasonable agreement with those calculated from TGA data using Equation 1. The latter data are considered to be more reliable.

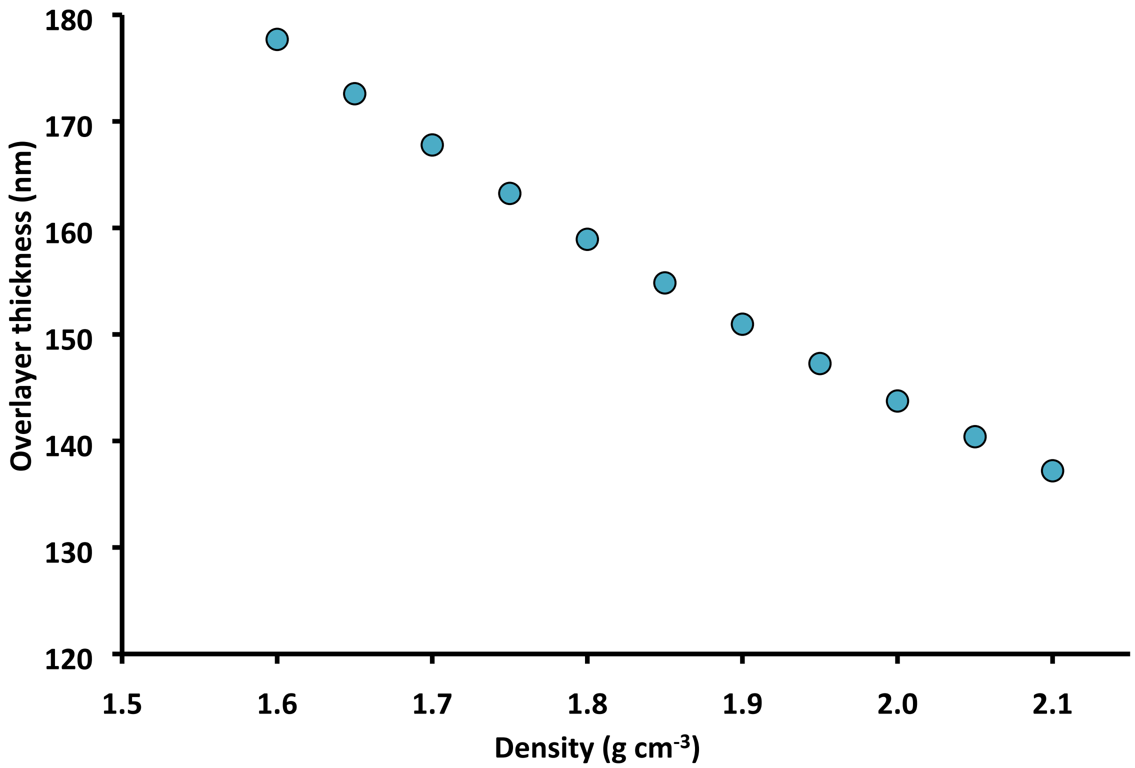

Figure S6. Variation in silica overlayer thickness as a function of silica overlayer density calculated using Equation 1 (assuming a mean PMMA latex radius of 3000 nm, a PMMA density of 1.206 g cm^{-3}, a PMMA core mass fraction of 0.80 and a silica shell mass fraction of 0.20).

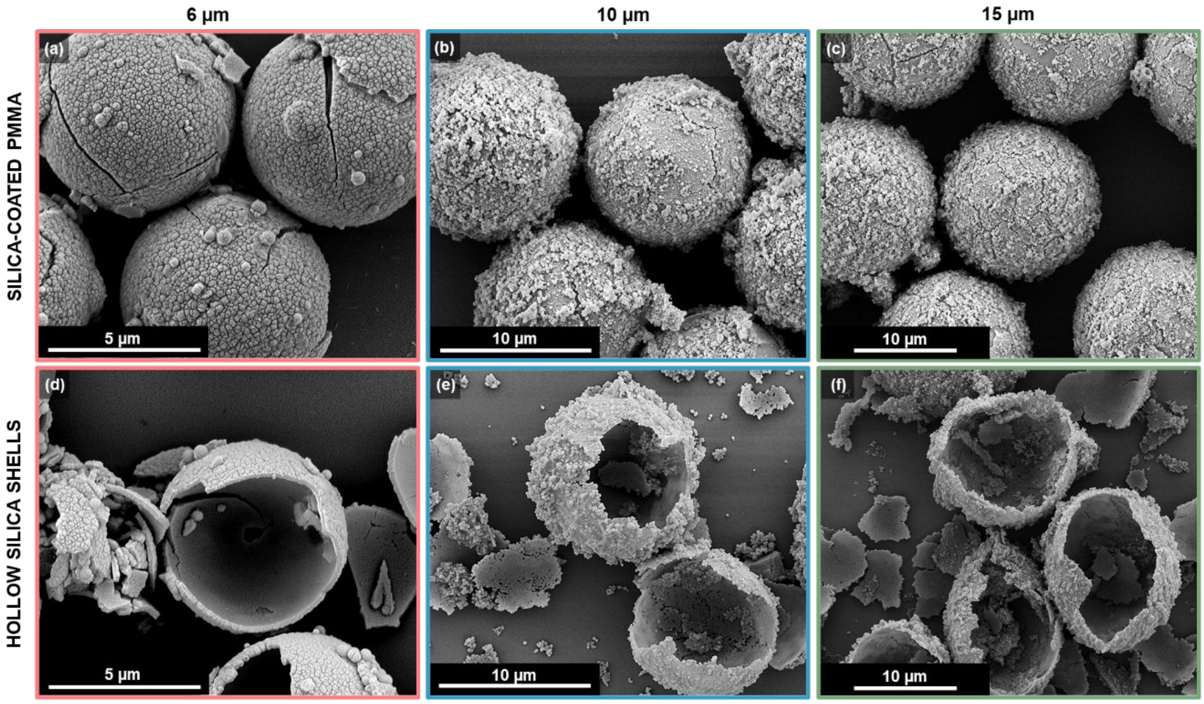

Figure S7. SEM images recorded for silica-coated PMMA latex particles with a mean diameter of (a) 6 µm, (b) 10 µm or (c) 15 µm when targeting an overlayer thickness of approximately 150 nm. The corresponding hollow silica shells after calcination were also recorded, see images (d-f).

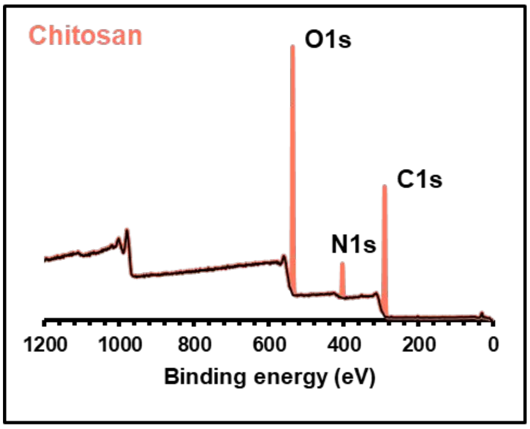

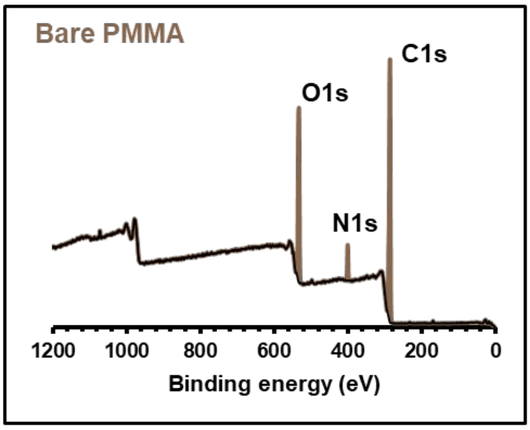

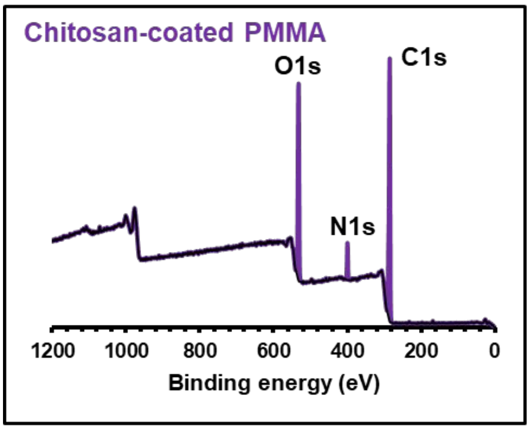

Sample	Signal	Intensity (%)
Chitosan	O1s	34.72
	N1s	7.56
	C1s	57.73
Bare PMMA	O1s	21.26
	N1s	4.27
	C1s	74.47
Chitosan-coated PMMA	O1s	22.8
	N1s	4.56
	C1s	72.65

Figure S8. XPS survey spectra recorded for chitosan alone, bare 6 µm PMMA latex and chitosan-coated 6 µm PMMA latex. The corresponding tabulated data is also provided.

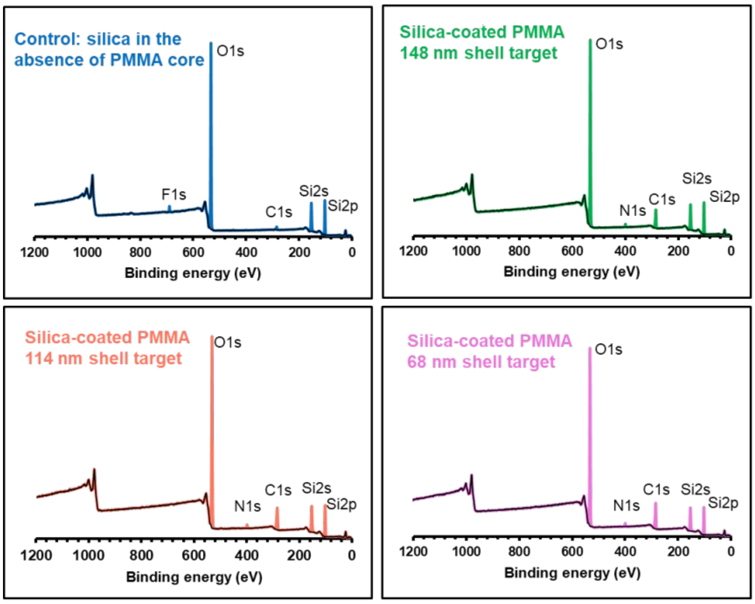

Sample	Signal	Intensity (%)
148 nm shell target	O1s	54.92
	N1s	1.46
	C1s	18.48
	Si2p	25.15
114 nm shell target	O1s	53.31
	N1s	1.47
	C1s	21.02
	Si2p	24.20
68 nm shell target	O1s	51.22
	N1s	1.56
	C1s	25.26
	Si2p	21.97
Control silica	O1s	64.93
	C1s	3.95
	Si2p	31.11

Figure S9. XPS survey spectra recorded for a silica reference sample prepared in the presence of chitosan but in the absence of any latex (blue spectrum) and three silica-coated 6 µm PMMA latexes when targeting shell thicknesses of 148 nm (green spectrum), 114 nm (orange spectrum) or 68 nm (pink spectrum). The corresponding tabulated data is also provided.
